# RORγt-Raftlin1 complex regulates the pathogenicity of Th17 cells and colonic inflammation

**DOI:** 10.1038/s41467-023-40622-1

**Published:** 2023-08-17

**Authors:** Amir Kumar Singh, Ritesh Kumar, Jianyi Yin, John F. Brooks II, Mahesh Kathania, Sandip Mukherjee, Jitendra Kumar, Kevin P. Conlon, Venkatesha Basrur, Zhe Chen, Xianlin Han, Lora V. Hooper, Ezra Burstein, K. Venuprasad

**Affiliations:** 1 Department of Internal Medicine, UT Southwestern Medical Center, Dallas, TX 75390 USA; 2 Department of Immunology, UT Southwestern Medical Center, Dallas, TX 75390 USA; 3 Harold C. Simmons Comprehensive Cancer Center, UT Southwestern Medical Center, Dallas, TX 75390 USA; 4 Department of Pathology, University of Michigan, Ann Arbor, MI 48109 USA; 5 Department of Biophysics, UT Southwestern Medical Center, Dallas, TX 75390 USA; 6 University of Texas Health Science Center at San Antonio, San Antonio, TX 78229 USA; 7 The Howard Hughes Medical Institute, UT Southwestern Medical Center, Dallas, TX 75390 USA

**Keywords:** Gastrointestinal diseases, Mucosal immunology, T-helper 17 cells, Inflammation

## Abstract

Th17 cells that produce Interleukin IL-17 are pathogenic in many human diseases, including inflammatory bowel disease, but are, paradoxically, essential for maintaining the integrity of the intestinal barrier in a non-inflammatory state. However, the intracellular mechanisms that regulate distinct transcriptional profiles and functional diversity of Th17 cells remain unclear. Here we show Raftlin1, a lipid raft protein, specifically upregulates and forms a complex with RORγt in pathogenic Th17 cells. Disruption of the RORγt-Raftlin1 complex results in the reduction of pathogenic Th17 cells in response to *Citrobacter rodentium;* however, there is no effect on nonpathogenic Th17 cells in response to commensal segmented filamentous bacteria. Mechanistically, we show that Raftlin1 recruits distinct phospholipids to RORγt and promotes the pathogenicity of Th17 cells. Thus, we have identified a mechanism that drives the pathogenic function of Th17 cells, which could provide a platform for advanced therapeutic strategies to dampen Th17-mediated inflammatory diseases.

## Abstract (web summary)

IL-17 secreting Th17 cells have established roles in numerous immune-pathologies but paradoxically are required homeostatically in the maintenance of the intestinal barrier. Here the authors establish Raftlin-1 in the recruitment of phospholipids that are linked to the emergence of pathogenic Th17 cells in animal models of bowel inflammation.

## Introduction

Interleukin (IL)−17 produced by Th17 cells plays a crucial role in the pathogenicity of several human inflammatory diseases^1–3^. However, not all Th17 cells are pathogenic because Th17 cells present in the gut mucosa are essential for tissue homeostasis by preventing invasion of gut microflora and promoting epithelial barrier functions^4^. Th17 cells also play an indispensable role in host defense against bacterial and fungal infections^5^. In addition, gut Th17 cells have been suggested to influence extra-intestinal inflammation since their microbiota-specific induction leads to exacerbated disease at extra-intestinal sites in multiple disease models^6–8^. However, the intracellular mechanisms that regulate distinct transcriptional profiles and functional diversity of Th17 cells remain unclear.

Although RORγt, a member of the nuclear receptor (NR) family of proteins, is well documented as a transcription factor for Th17 cells^3,9^, it remains unclear how RORγt drives the distinct transcriptional profiles seen in pathogenic vs. nonpathogenic Th17 cells. RORγt is composed of an N-terminal ligand-independent activation function 1 (AF1) domain, a DNA-binding domain (DBD), a flexible hinge region, and a C-terminal ligand-binding domain (LBD)^9^. The NR family of transcription factors are activated by the binding of specific ligands to their LBD domain^3^. The ligands of NRs are mostly hydrophobic molecules that are derivatives of cholesterol, fatty acids, retinoids, vitamins, and lipophilic hormones^10^. It remains unclear if RORγt function is regulated by its natural ligands, although evidence suggests the existence of such ligands^11,12^.

Here, we demonstrate that Raftlin1, a lipid raft protein, directly associates with RORγt and recruits specific lipids to modulate the pathogenicity of Th17 cells.

## Results

### Raftlin1 is upregulated in pathogenic Th17 cells and associates directly with RORγt

**Fig. 1:**
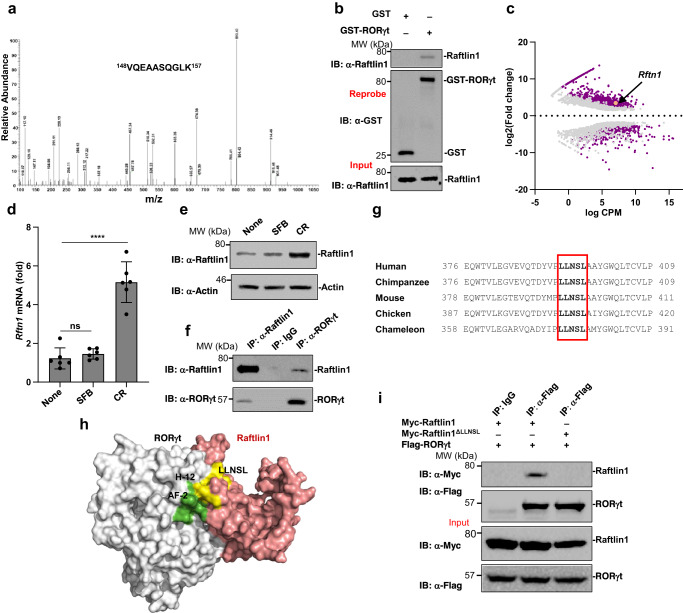
Raftlin1 is upregulated in pathogenic Th17 cells and binds to RORγt. **a** MS spectrum of peptide ^148^VQEAASQGLK^157^ corresponding to Raftlin1 (UniProt accession No: Q6A0D4). Observed b- and y-ions are indicated. **b** The lysates from CD4^+^ T cells isolated from colonic lamina propria of *C. rodentium* infected WT mice were pulled-down with purified recombinant GST or GST-RORγt followed by Immunoassays with anti-Raftlin1 and anti-GST antibody. **c** GEO dataset (GSE130302) was analyzed (*n* = 2 *C. rodentium-*infected and *n* = 3 SFB-colonized colon) and presented as the MA plot showing the change in expression (log2 fold change) and counts per million (log CPM values); *p* < 0.05, FDR < 0.05. **d** *Rftn1* mRNA expression was analyzed by real-time PCR using CD4^+^ T cells isolated from colonic lamina propria; *n* = 6 mice per group. Data are presented as mean value ± SD, *p* = 0.00010 (CR), *p* = 0.37 (SFB). **e** Immunoassays of Raftlin1 protein in the lysates of CD4^+^ T cells. **f** the lysates of CD4^+^ T cells isolated from colonic lamina propria of *C. rodentium* infected mice were subjected to immunoprecipitation with anti-Raftlin1 or anti-RORγt antibody and immunoblot analysis with anti-RORγt or anti-Raftlin1 antibody. **g** Sequence alignment of Raftlin1; box indicates the conserved LLNSL motif. **h** Putative protein-protein interface from the docking model is shown in color gray for RORγt and Salmon for Raftlin1. Helix 12 (labeled) and AF2 (green) domains of RORγt are involved in non-covalent interaction in the modeled RORγt-Raftlin1 complex. LLNSL region of Raftlin1 is shown in color yellow. **i** Lysates from HEK293T cells transfected with various combinations of plasmids encoding Flag-RORγt and either Myc-Raftlin1 or Myc-Raftlin1^ΔLLNSL^, followed by immunoprecipitation with anti-Flag antibody and immunoblot analysis with anti-Myc or anti-Flag antibody. Immunoblots in (**b**, **e**, **f**, **i**) are from one experiment representative of three independent experiments with similar results. Statistical significance was determined by unpaired Student’s *t* test (two-tailed) in (**c**, **d**) with *p*  <  0.05 considered statistically significant. ****p  <  0.0001; ns- not significant. Source data are provided as a Source data file.

*C. rodentium*, a Gram-negative extracellular bacterial pathogen of mice akin to the human enterohemorrhagic *Escherichia coli*, elicits pathogenic Th17 cells in the gastrointestinal tract^13^. To gain molecular insights into the regulation of RORγt in pathogenic Th17 cells, we inoculated approximately 2 × 10^9^ colony-forming units of *C. rodentium* through oral gavage to C57BL/6 (WT) mice. We isolated CD4^+^ T cells from colonic lamina propria and performed immunoprecipitation assays with anti-RORγt antibody using cell lysates. The precipitated proteins were subjected to mass spectrometry (MS) analysis. We identified Raftlin1 as a RORγt binding protein in precipitates of anti-RORγt antibody but not in IgG controls. A representative MS spectrum corresponding to ^148^VQEAASQGLK^157^ is shown in Fig. 1a. We confirmed the MS results by performing GST-Pull down assays. The lysates from CD4^+^ T cells isolated from colonic lamina propria of *C. rodentium* infected mice were pulled down with purified recombinant GST-only or GST-RORγt protein and immunoblotted with anti-Raftlin1 antibody. In immunoblot assays, GST-RORγt protein was able to interact with Raftlin1 but not the GST protein alone (Fig. 1b).

Raftlin1 was originally identified as a lipid raft protein in B cells^14^ and was later shown to be involved in experimental autoimmune encephalomyelitis (EAE), a Th17-dependent autoimmune disease model^15^. Since our results showed an association of RORγt with Raftlin1 in CD4^+^ T cells isolated from colonic lamina propria of mice infected with *C. rodentium*, we analyzed a published^16^ RNA-seq data set (GSE130302) to test if the level of Raftlin1 expression altered following infection. Interestingly, *Rftn1* was selectively upregulated in *C. rodentium* infected but not SFB monocolonized mice (Fig. 1c). To confirm these results, we performed real-time PCR with *Rftn1*-specific primers from RNA and immunoblot assays in CD4^+^ T cells isolated from colonic lamina propria of *C. rodentium* infected mice showing that Raftlin1 expression was upregulated in CD4^+^ T cells isolated from the colonic lamina propria of *C. rodentium* infected mice (Fig. 1d, e). We also tested if Raftlin2, an isoform of Raftlin1, expression is similarly altered. However, no significant difference in *Rftn2* expression was observed (Supplementary Fig. 1a). Next, we analyzed the RORγt-Raftlin1 association by performing a co-immunoprecipitation experiment using the lysate from CD4^+^ T cells isolated from the colonic lamina propria of *C. rodentium* infected mice, and SFB colonized mice. Anti-RORγt immunoprecipitated Raftlin1 and anti-Raftlin1 immunoprecipitated RORγt in *C. rodentium* infected but not SFB colonized mice (Fig. 1f and Supplementary Fig. 1b). Together, these data suggest that Raftlin1 is upregulated and associated with RORγt following *C. rodentium* infection.

### Raftlin1 associates with RORγt through the LLNSL motif

Most NRs bind to their coactivators/corepressors containing LXXLL (L-leucine, X- any amino acid) motifs through their AF2 domain^17^. Interestingly, the sequence alignment of Raftlin1 revealed a highly conserved LLNSL motif which could bind to the AF2 domain of RORγt (Fig. 1g). Further, docking of Raftlin1 with the ligand binding domain (LBD) of RORγt suggested that the conserved LLNSL motif of Raftlin1 forms noncovalent interactions with the AF2 domain of RORγt (Fig. 1h, Supplementary Fig. 1c). Also, deletion of LLNSL completely disrupted the protein-protein interface between RORγt and Raftlin1 (Supplementary Fig. 1d), further supporting the notion that Raftlin1 binds to RORγt via the LLNSL motif. To experimentally verify this observation, we deleted the LLNSL motif of Raftlin1 (Raftlin1^ΔLLNSL^). Co-immunoprecipitation experiments showed wild-type Raftlin1 but not Raftlin1^ΔLLNSL^ associated with RORγt (Fig. 1i). These data collectively suggest that Raftlin1 associates with RORγt via the LLNSL motif.

### Raftlin1 transactivates RORγt

**Fig. 2:**
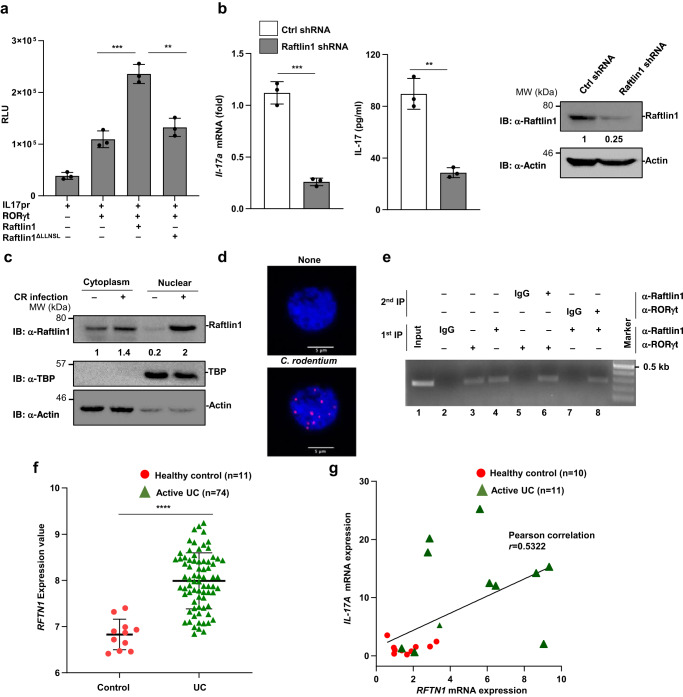
Raftlin1 regulates IL-17 expression, and the level of Raftlin1 expression correlates with IL-17 expression in UC patients. **a** Luciferase assay of lysates of Jurkat T cells transfected with various plasmid combinations (below plot) of the IL-17 promoter-driven luciferase plasmid (pGL4-IL17pr) and the plasmid encoding Flag-RORγt along with wild-type Myc-Raftlin1 or Myc-Raftlin1^ΔLLNSL^; results are presented in relative luciferase unit (RLU); *n* = 3 biological replicates, *p* = 0.0009, *p* = 0.002. **b** Real-time PCR and ELISA were performed to check the expression of IL-17 in Raftlin1 knockdown lysates of CD4^+^ T cells isolated from colonic lamina propria of *C. rodentium* infected WT mice; *n* = 3 biological replicates, *p* = 0.0002 (*Il-17a*), *p* = 0.001 (IL-17a). Immunoblot showing knockdown of Raftlin1. **c** Immunoblot analysis for Raftlin1 in the cytoplasmic and nuclear fraction of CD4^+^ T cells isolated from colonic lamina propria. **d** Representative images from proximity ligation assay (PLA) showing the interaction between RORγt and Raftlin1. The image is representative of three independent experiments. Scale bars, 5 μm. **e** Binding of RORγt-Raftlin1 to the IL-17 promoter sites was assessed by ChIP, and re-ChIP analysis using a DNA-protein complex using a specific antibody. **f** GEO dataset (GSE59071) was analyzed (*n* = 11 healthy control and *n* = 74 active UC patients samples), and the normalized expression value of *RFTN1* is as shown above, *p* = 2 × 10^−9^. **g** Expression of *IL-17A* mRNA and *RFTN1* mRNA was analyzed by real-time PCR in active UC and control samples (*n* = 10 healthy control and *n* = 11 active UC patients samples). Relative *IL-17A* mRNA was plotted against relative *RFTN1* mRNA; *p* = 0.0130, Pearson correlation coefficient *r* = 0.5322. Data in (**a–c**, **e**) are from one experiment representing three independent experiments with similar results. Statistical significance was determined by unpaired Student’s *t* test (two-tailed) in (**a**, **b**, **f**, **g**) with *p*  <  0.05 considered statistically significant. **p  <  0.01; ***p  <  0.001; ****p  <  0.0001, error bars are mean ± SD. The statistics (g) were measured by the Pearson correlation coefficient. Source data are provided as a Source data file.

To gain insights into the functional consequence of the RORγt-Raftlin1 interaction, we performed an IL-17-promoter-driven luciferase assay^18^. As shown in Fig. 2a, co-expression of wild-type Raftlin1 but not Raftlin1^ΔLLNSL^ and RORγt enhanced luciferase activity. Further, knockdown of Raftlin1 using shRNA expressing lentiviral constructs in CD4^+^ T cells isolated from the colonic lamina propria significantly decreased IL-17 expression compared to control (Fig. 2b). These results suggest that inhibition of Raftlin1 attenuates IL-17 expression.

Next, we analyzed the cellular localization of Raftlin1 following *C. rodentium* infection in WT mice. We separated cytoplasmic and nuclear fractions of CD4^+^ T cells isolated from the colonic lamina propria, followed by immunoblotting. We detected an increased accumulation of Raftlin1 in the nuclear fraction of CD4^+^ T cells isolated from the colonic lamina propria (Fig. 2c). Proximity ligation assays showed colocalization of Raftlin1 with RORγt in the nucleus of CD4^+^ T cells isolated from the colonic lamina propria of *C. rodentium* infected mice (Fig. 2d). Altogether, these results suggest that Raftlin1 translocated to the nucleus and formed a close interaction with RORγt following infection with *C. rodentium*.

To test if the RORγt-Raftlin1 complex binds to the IL-17 promoter, we performed sequential chromatin immunoprecipitation (ChIP) assays. Sonicated nuclei from formaldehyde-treated CD4^+^ T cells isolated from the colonic lamina propria of *C. rodentium* infected mice as we described before^19^. The immunoprecipitated (IP’d) DNA-protein complexes were eluted, and further IP’d using the opposite antibody. As shown in Fig. 2e lanes 3 and 4, Raftlin1 was detected in both Raftlin1 and RORγt-IP’d chromatin, suggesting a strong association of RORγt and Raftlin1 with the IL-17 promoter. The interaction between RORγt and Raftlin1 to the IL-17 promoter was further confirmed by re-ChIP analysis. The DNA-protein complexes were first IP’d using reciprocal anti-RORγt or anti-Raftlin1 antibody, and the pulled-down immunocomplex was eluted, and further IP’d using anti-Raftlin1 or anti-RORγt antibody. As shown in Fig. 2e lanes 6 and 8, the interaction of RORγt and Raftlin1 was found to be associated with the IL-17 promoter. Together, these results suggest that the RORγt-Raftlin1 complex binds to the IL-17 promoter.

### Raftlin1 expression correlates with IL-17 level in ulcerative colitis patients

Next, we investigated *RFTN1* expression in ulcerative colitis (UC) patients. Analysis of published RNA-Sequencing data (GSE 59071)^20^ showed that the gene expression of *RFTN1* was preferentially upregulated in active UC (Fig. 2f) and Crohn’s disease patient samples (Supplementary Fig. 2). Previous studies have demonstrated elevated levels of IL-17 in the mucosa of UC patients and was shown to contribute to mucosal inflammation and significant tissue damage^21^. Since our results showed that Raftlin1 interacts with RORγt and increases IL-17 activity, we investigated whether *RFTN1* expression levels in CD4^+^ T cells correlated with *IL-17A* activity in active UC patients. In real-time PCR experiments using RNA isolated from CD4^+^ T cells isolated from the colonic biopsy samples^22^ of active UC patients, we observed that mRNA expression of both *RFTN1* and *IL-17A* was elevated in a significant number of active UC patients. As shown in Fig. 2g, there was a positive correlation (Pearson coefficient, *r* = 0.53) between *RFTN1* mRNA expression and *IL-17A* levels. These data show the clinical importance of Raftlin1-mediated regulation of Th17 cells in UC patients.

### Reduced pathogenic Th17 cells in Raftlin1^ΔLLNSL^ mice

**Fig. 3:**
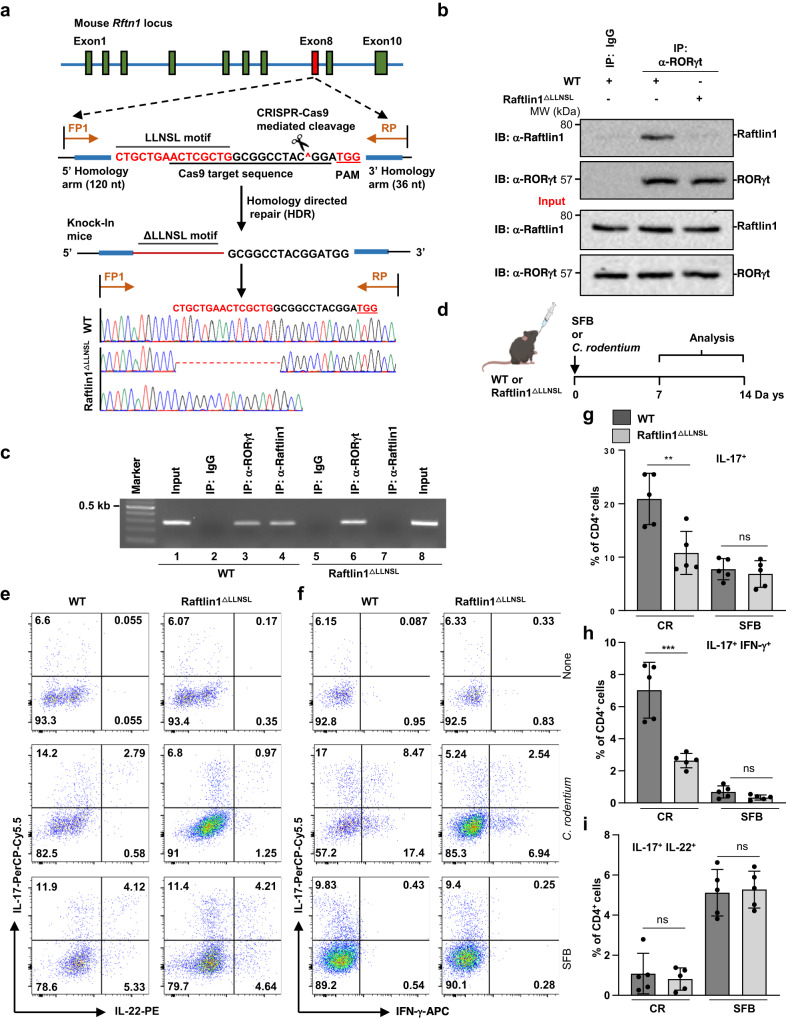
Disruption of RORγt-Raftlin1 interaction attenuates pathogenic but not nonpathogenic Th17 responses. **a** Generation of Raftlin1 knock-in (Raftlin1^∆LLNSL^) mice using the CRISPR-Cas9 approach. Schematic illustration of the *Rftn1* gene structures, sequences around the target locus, and donor oligos template (in the “Methods” sections). The sequence around the target locus indicates the PAM and the sequence recognized by the Cas9-gRNA complex. The Raftlin1 knock-in mice were confirmed by genotyping using PCR and Sanger sequencing. **b** Lysates from CD4^+^ T cells isolated from colonic lamina propria of *C. rodentium* infected WT and Raftlin1^∆LLNSL^ mice followed by immunoprecipitation with anti-RORγt antibody and immunoblot analysis with anti-Raftlin1 antibody. **c** Binding of RORγt-Raftlin1 complex to the IL-17 promoter sites was assessed by ChIP analysis using a DNA-protein complex from CD4^+^ T cells isolated from colonic lamina propria of *C. rodentium* infected WT and Raftlin1^∆LLNSL^ mice. **d** Schematic diagram of the experimental procedure for *C. rodentium* and SFB-induced generation of Th17 cells. **e**, **f** Representative image of colonic lamina propria cells stained with antibodies against CD4, and intracellularly for IL-17, IFN-γ and IL-22, and analyzed by flow cytometry; *n* = 5 mice per group. **g–i** Corresponding quantification of total IL17^+^, IL-17^+^ IFN-γ^+^ and IL-17^+^ IL-22^+^ cells in CD4^+^ cells from colonic lamina propria cells of WT and Raftlin1^∆LLNSL^ mice infected with *C. rodentium* or colonized with SFB; *n* = 5 mice per group, IL17^+^: *p* = 0.007 (WT and Raftlin1^∆LLNSL^ mice with CR), *p* = 0.544 (WT and Raftlin1^∆LLNSL^ mice with SFB); IL-17^+^IFN-γ^+^: *p* = 0.0006 (WT and Raftlin1^∆LLNSL^ mice with CR), *p* = 0.089 (WT and Raftlin1^∆LLNSL^ mice with SFB); IL-17^+^IL-22^+^: *p* = 0.616 (WT and Raftlin1^∆LLNSL^ mice with CR), *p* = 0.815 (WT and Raftlin1^∆LLNSL^ mice with SFB). **b**, **c** Data are from one experiment which is representative of three independent experiments with similar results. Statistical significance was determined by unpaired Student’s *t* test (two-tailed) in (**g–i**) with *p*  <  0.05 considered statistically significant. **p  <  0.01; ***p  <  0.001; ns- not significant, error bars are mean ± SD. Source data are provided as a Source data file. The figure (**d**) was created using BioRender (https://biorender.com/).

To further investigate the function of the RORγt-Raftlin1 complex in the regulation of gastrointestinal inflammation, we generated Raftlin1 knock-in (Raftlin1^ΔLLNSL^) mice lacking the LLNSL motif using the CRISPR-Cas9 approach. Resultant pups were screened for the *Rftn1* knock-in region at exon 8 by PCR and Sanger sequencing (Fig. 3a, Supplementary Fig. 3a). The potential off-target sites of gRNA in Raftlin1^∆LLNSL^ mice were checked by Sanger sequencing (Supplementary Fig. 3b). The Raftlin1^ΔLLNSL^ mice developed and bred normally and showed no sign of overt inflammatory disorders. The number of CD4^+^, CD8^+^, Foxp3^+^ and CD25^+^ T cells in the thymus, spleen, lymph nodes, and Peyer’s patches in Raftlin1^ΔLLNSL^ mice was comparable to WT littermate control mice (Supplementary Fig. 4a–e). Similarly, the number of naïve and memory T cells was similar (Supplementary Fig. 4f, g).

The expression of Raftlin1 was similar in WT and Raftlin1^ΔLLNSL^ mice after *C. rodentium* infection (Supplementary Fig. 5a, b). Also, WT and Raftlin1^ΔLLNSL^ Th17 cells expressed RORγt normally (Supplementary Fig 5c). Raftlin1 has been shown to be involved in protein trafficking and is essential for the formation of lipid rafts^14,15,23^. Therefore, we tested if the deletion of the LLNSL motif from Raftlin1 affected the formation of lipid rafts in T cells using PE-labeled Cholera toxin B and found no defect in the formation of lipid rafts in Raftlin1^∆LLNSL^ T cells (Supplementary Fig. 5d). Additionally, localization of Raftlin1 in WT and Raftlin1^ΔLLNSL^ CD4^+^ T cells were similar (Supplementary Fig. 5e). Also, recruitment of Lck to the TCR complex and phosphorylation of Zap70 and IκBα were found to be normal (Supplementary Fig. 5f, g). Further, the expression of CD25 and *Il-2* following stimulation with anti-CD3 and anti-CD28 antibodies was comparable to WT T cells (Supplementary Fig. 5h, i). Also, deletion of the LLNSL motif from Raftlin1 did not affect the differentiation of T cells into Th1 and Th2 but significantly attenuated IL-17 expression by Th17 cells (Supplementary Fig. 5j). These data collectively suggested that deletion of the LLNSL motif did not overtly affect thymic development and early TCR signaling in T cells. To investigate the effect of deletion of the LLNSL motif on RORγt-Raftlin1 interaction in CD4^+^ T cells isolated from colonic lamina propria, we performed a co-immunoprecipitation experiment. Our results showed that RORγt was associated with Raftlin1 only in CD4^+^ T cells isolated from colonic lamina propria of *C. rodentium* infected WT but not in Raftlin1^ΔLLNSL^ mice (Fig. 3b). This confirmed that Raftlin1 associates with RORγt in vivo via the LLNSL motif.

Next, we investigated whether deletion of the LLNSL motif of Raftlin1 affected the binding of the RORγt-Raftlin1 complex to the IL-17 promoter. We performed ChIP assays using CD4^+^ T cells isolated from colonic lamina propria of WT and Raftlin1^ΔLLNSL^ mice infected with *C. rodentium*. As shown in Fig. 3c, WT Raftlin1 but not Raftlin1^ΔLLNSL^ binds to the IL-17 promoter, confirming that Raftlin1 complexes with RORγt through the LLNSL motif and binds to the IL-17 promoter. Next, we evaluated in vivo Th17 response in Raftlin1^ΔLLNSL^ mice. Commensals such as SFB and intestinal pathogens like *C. rodentium* induce distinct Th17 responses. SFB colonization promotes a nonpathogenic Th17 response in the intestinal lamina propria, which predominantly involves IL-17 and IL-22^24^, whereas Th17 cells elicited by *C. rodentium* produce IL-17 and IFN-γ and are pathogenic^16^. Since our results showed increased expression of Raftlin1 in CD4^+^ T cells isolated from colonic lamina propria of mice infected with *C. rodentium* and formed a complex with RORγt (Fig. 1e, f), we tested the effect of disruption of the RORγt-Raftlin1 interaction on the generation of pathogenic vs. nonpathogenic Th17 cells. We gavaged littermate WT control and Raftlin1^ΔLLNSL^ mice with fecal samples of mice monocolonized with SFB (Fig. 3d)^25^. We isolated bacterial DNA from the fecal pellets and analyzed SFB colonization by real-time PCR using SFB 16 S rRNA gene sequence-specific primers^25^. Our results showed that SFB colonization was similar between WT and Raftlin1^ΔLLNSL^ mice throughout the experiment (Supplementary Fig. 6a). In the case of *C. rodentium*, we observed similar bacterial load at early time points (at day 8) in the feces of WT and Raftlin1^ΔLLNSL^ mice. However, at later stages, from day 9, an increased abundance of *C. rodentium* was observed in the feces of Raftlin1^ΔLLNSL^ mice compared to WT mice (Supplementary Fig. 6b). Flow cytometry analysis showed a similar number of IL-17^+^IL-22^+^ nonpathogenic Th17 cells in WT and Raftlin1^ΔLLNSL^ mice (Fig. 3e–i). Since the presence of SFB in mouse facilities could affect the outcome of these experiments^24^, real-time PCR analysis of 16 S rRNA using the DNA isolated from the fecal pellets of our mouse colony showed it to be devoid of SFB (Supplementary Fig. 6c). Next, we gavaged Raftlin1^ΔLLNSL^ and littermate WT control mice with *C. rodentium*, and our flow cytometry analysis showed a reduced number of IL-17^+^IFN-γ^+^ pathogenic Th17 cells in Raftlin1^ΔLLNSL^ mice (Fig. 3e–I, Supplementary Fig. 7a). These data suggest that the RORγt-Raftlin1 complex plays an essential role in the generation of pathogenic Th17 cells.

**Fig. 4:**
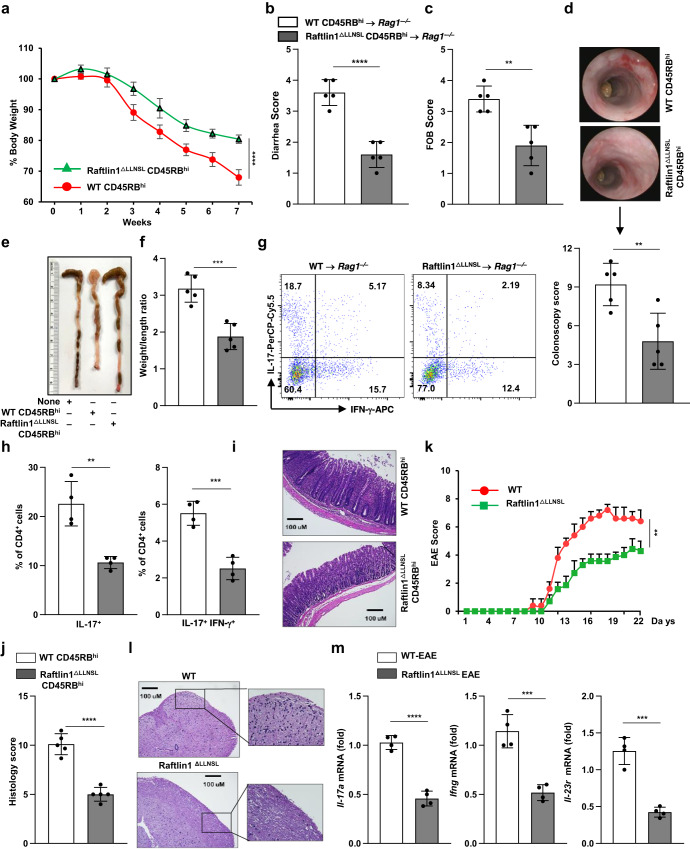
Deletion of the LLNSL motif of Raftlin1 attenuates the pathogenicity of Th17 cells. CD4^+^CD25^–^CD45RB^hi^ cells were FACS sorted from WT and Raftlin1^∆LLNSL^ mice and were adoptively transferred to *Rag1*^*–/–*^ mice. **a** Percent change in body weight; *n* = 5 mice per group, *p* = 0.00010. **b**, **c** Diarrhea score and fecal occult blood (FOB) score; *n* = 5 mice per group, *p* = 0.00006 (diarrhea score), *p* = 0.0025 (FOB). **d** Colonoscopy images and scores; *n* = 5 mice per group, *p* = 0.0068. **e** Representative image of colon length. **f** Colon weight/length ratio; *n* = 5 mice per group, *p* = 0.0005. **g** Representative image of colonic lamina propria cells stained with antibodies against CD4, and intracellularly for IL-17 and IFN-γ, and analyzed by flow cytometry; *n* = 4 mice per group. **h** Corresponding quantification of total IL17^+^ and IL-17^+^ IFN-γ^+^ in CD4^+^ cells from colonic lamina propria cells of *Rag1*^*–/–*^ mice; *n* = 4 mice per group, *p* = 0.0022 (IL17^+^), *p* = 0.0005 (IL-17^+^ IFN-γ^+^). **i** Microscopic images of H&E-stained colonic sections. The image is representative of five independent experiments. Scale bars, 100 μm. **j** Histology scores; *n* = 5 mice per group, *p* = 0.00002. **k** WT, and Raftlin1^ΔLLNSL^ mice were immunized with MOG_35-55_ peptide in complete Freund’s adjuvant. Clinical EAE scores (0–10 severity scale); *n* = 5 WT and 7 Raftlin1^∆LLNSL^ mice, *p* = 0.0013. **l** H&E-stained sections of the spinal cord. Image is representative of four independent experiments. Scale bars, 100 μm. **m** Expression of *Il-17a*, *Ifng*, and *Il-23r* were analyzed by real-time PCR in those spinal cords; *n* = 4 mice per group, *p* = 0.00003 (*Il-17a*), *p* = 0.0005 (*Ifng*), and *p* = 0.0001 (*Il-23r*). Data are from one experiment representative of three independent experiments with similar results. Statistical significance was determined by unpaired Student’s *t* test (two-tailed) in (**a–d**, **f**, **h**, **j**, **k**, **m**) with *p*  <  0.05 considered statistically significant. **p  <  0.01; ***p  <  0.001; ****p  <  0.0001, error bars are mean ± SD. Source data are provided as a Source data file.

To further confirm the function of the RORγt-Raftlin1 complex in Th17-mediated pathogenic inflammation, we utilized an adoptive transfer colitis model^26,27^. We sorted CD4^+^CD25^−^CD45RB^hi^ cells from WT and Raftlin1^ΔLLNSL^ mice which were adoptively transferred into *Rag1*^*–/–*^ mice^18^. *Rag1*^*–/–*^ mice that received Raftlin1^ΔLLNSL^ T cells showed reduced loss of body weight, lower fecal occult blood, and diarrhea scores (Fig. 4a–c) as compared to mice that received WT T cells. *Rag1*^*–/–*^ mice exhibited less inflammation in the colon, reduced spleen size, and less shortening of the colon compared to mice that received WT T cells (Fig. 4d–f). Further, *Rag1*^*–/–*^ mice that received Raftlin1^∆LLNSL^ T cells exhibited a reduced number of IL-17^+^IFN-γ^+^ cells in the colonic mucosa (Fig. 4g, h, Supplementary Fig. 7b). Histologic analysis of colonic tissue from these mice showed reduced infiltration of inflammatory cells, less crypt damage, and lower disease scores (Fig. 4i, j).

To test the effect of disruption of the RORγt-Raftlin1 complex in regulating inflammation at extra-intestinal sites, we extended our studies to experimental autoimmune encephalomyelitis (EAE), a mouse model of multiple sclerosis^28^. We immunized WT mice and Raftlin1^ΔLLNSL^ mice with the myelin oligodendrocyte glycoprotein (MOG_35-55_) peptide in complete Freund’s adjuvant to induce EAE^28^. Raftlin1^ΔLLNSL^ mice developed EAE slower and with less severity than WT mice, as indicated by disease scores (Fig. 4k, l). Further, Raftlin1^∆LLNSL^ mice showed reduced expression of *Il-17a*, *Ifng*, and *Il-23r* in their spinal cord (Fig. 4m). Together, these findings suggest that the RORγt-Raftin1 complex plays an essential role in the pathogenesis of Th17 cells.

### Raftlin1 recruits phospholipids to RORγt and promotes the pathogenicity of Th17 cells

**Fig. 5:**
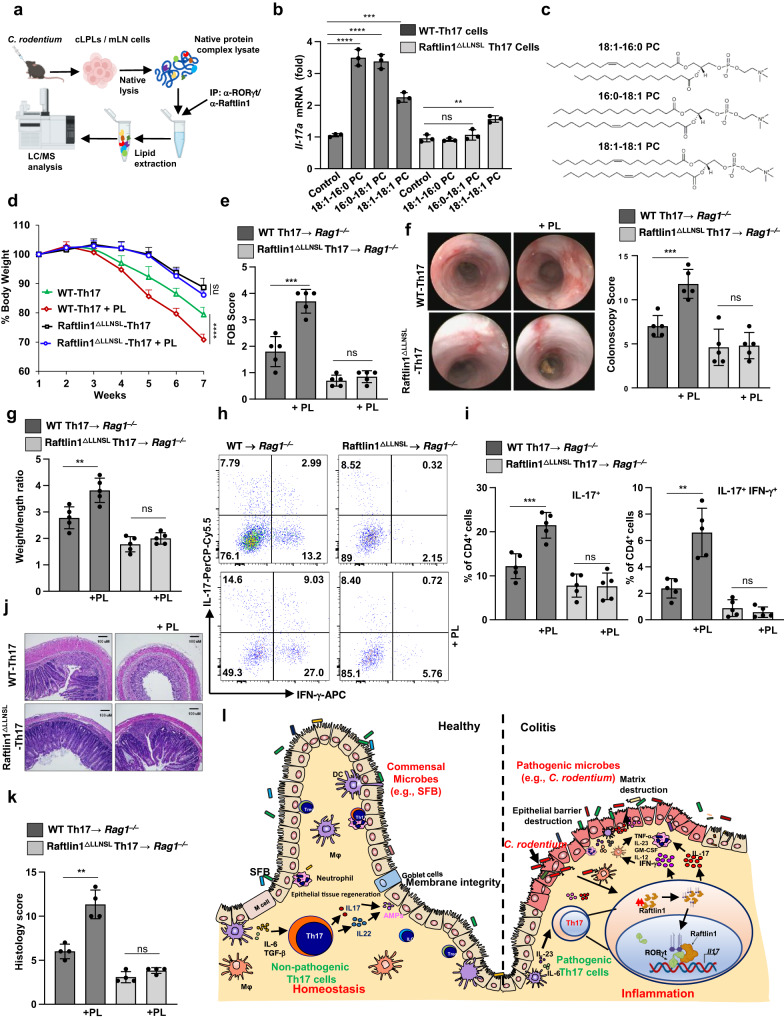
Raftlin1 recruits phospholipids to RORγt and promotes the pathogenicity of Th17 cells. **a** Schematic representation of the lipidomics by LC/MS. **b** Naive CD4^+^ T cells were isolated from WT or Raftlin1^∆LLNSL^ mice and differentiated under Th17 polarizing conditions in the presence or absence of phospholipids (PL). *Il-17a* expression was measured by qPCR; *n* = 3 mice per group, *p* = 0.00008 (WT-Th17 cells with 18:1–16:0-PC), *p* = 0.00006 (WT-Th17 cells 16:0–18:1-PC), *p* = 0.0002 (WT-Th17 cells 18:1–18:1-PC), *p* = 0.389 (Raftlin1^∆LLNSL^ Th17 with 16:0–18:1-PC), *p* = 0.0022 (Raftlin1^∆LLNSL^ Th17 with 18:1–18:1-PC). **c** Structure of phospholipids. **d** Th17 cells were generated in the presence of PL. The cells were then adoptively transferred to *Rag1*^*–/–*^ mice. Percent change in body weight is shown. *p* = 0.00001 (WT and WT-PL), *p* = 0.056 (Raftlin1^∆LLNSL^ and Raftlin1^∆LLNSL^ with PL). **e** Fecal occult blood score. *p* = 0.0004 (WT and WT-PL), *p* = 0.305 (Raftlin1^∆LLNSL^ and Raftlin1^∆LLNSL^ with PL). **f** Colonoscopy images, and scores. *p* = 0.0008 (WT and WT-PL), *p* = 0.865 (Raftlin1^∆LLNSL^ and Raftlin1^∆LLNSL^ with PL). **g** Colon weight/length ratio. *p* = 0.0056 (WT and WT-PL), *p* = 0.20 (Raftlin1^∆LLNSL^ and Raftlin1^∆LLNSL^ with PL). **h** Representative image of colonic lamina propria cells stained with antibodies against CD4, intracellularly for IL-17 and IFN-γ and analyzed by flow cytometry. **i** Corresponding quantification of total IL-17^+^ and IL-17^+^IFN-γ^+^ cells. IL-17^+^: *p* = 0.0009 (WT and WT-PL), *p* = 0.93 (Raftlin1^∆LLNSL^ and Raftlin1^∆LLNSL^ with PL); IL-17^+^ IFN-γ^+^: *p* = 0.0014 (WT and WT-PL), *p* = 0.381 (Raftlin1^∆LLNSL^ and Raftlin1^∆LLNSL^ with PL). **j** Microscopic images of H&E-stained colonic sections. The image is representative of four independent experiments. Scale bars, 100 μm. **k** Histology score of the sections. *p* = 0.0011 (WT and WT-PL), *p* = 0.080 (Raftlin1^∆LLNSL^ and Raftlin1^∆LLNSL^ with PL). **l** The proposed model of RORγt-Raftlin1 complex in the regulation of pathogenicity of Th17 cells. **d–h**, **i**, *n* = 5 mice per group, (**k**) *n* = 4 mice per group. Statistical significance was determined by unpaired Student’s *t* test (two-tailed) in (**b**, **d–g**, **i**, **k**) with *p*  <  0.05 considered statistically significant. **p  <  0.01, ***p  <  0.001; ****p  <  0.0001, ns- not significant, error bars are mean ± SD. Source data are provided as a Source data file. Figures (**a**, **i**) were created using BioRender (https://biorender.com/).

We next sought to investigate the mechanism by which Raftlin1 modulates the pathogenicity of Th17 cells. Since our results showed that Raftlin1, which is a lipid raft protein, binds to the AF2 domain within the LBD domain of RORγt, we hypothesized that Raftlin1 recruits specific lipid ligands that drive the pathogenicity of Th17 cells. To test this hypothesis, we isolated mesenteric lymph node cells from *C. rodentium*-infected WT mice and cell lysates prepared in the absence of detergents^12^ followed by immunoprecipitation of RORγt and Raftlin1 using specific antibodies. The lipid species co-precipitated with RORγt and Raftlin1 were analyzed by LC-MS (Fig. 5a)^12^. A few lipids were consistently detected in the IP’s of both RORγt and Raftlin1 but not in the isotype controls (Supplementary Table 1). To identify the specific lipid(s) that promote RORγt transcriptional activity, we screened the identified lipids in an IL-17 promoter-driven luciferase assay. Co-expression of RORγt with Raftlin1 in Jurkat T cells cultured with the lipids showed that three phospholipids [1-oleoyl-2-palmitoyl-sn-glycero-3-phosphocholine (18:1–16:0-PC), 1-Palmitoyl-2-oleoyl-sn-glycero-3-phosphocholine (16:0–18:1-PC) and 1, 2-dioleoyl-sn-glycero-3-phosphatidylcholine (18:1–18:1-PC)] enhanced RORγt driven IL-17 promoter activity (Supplementary Fig. 8a). Next, we cultured naïve CD4^+^ T cells from WT and Raftlin1^∆LLNSL^ mice under Th17-inducing conditions in the presence of 18:1–16:0-PC, 16:0–18:1-PC, and 18:1–18:1-PC. Real-time PCR analysis showed increased *Il-17a* and *Il-23r* expression in WT but not Raftlin1^∆LLNSL^ Th17 cells generated in the presence of phospholipids (Fig. 5b, c, Supplementary Fig. 8b). To rule out the possibility of the effect of mutation of the LLNSL motif on localization of Raftlin1, we performed confocal microscopy analysis. We did not notice any significant change in the cellular distribution of WT or Raftlin1^∆LLNSL^ in CD4^+^ T cells isolated from the colonic lamina propria of *C. rodentium* infected mice (Supplementary Fig. 5e). Also, the presence or absence of phospholipids did not change the pattern of localization of WT Raftlin1 in Th17 cells (Supplementary Fig. 8c).

To validate the binding of phospholipids and RORγt, we chose a high-efficiency fluorescence resonance energy transfer (FRET) pair, donor 5-FAM (5-Carboxyfluorescein) labeled RORγt and acceptor Cyanine 5 (Cy5) labeled 1, 2-dioleoyl-sn-glycero-3-phosphatidylcholine (18:1–18:1-PC). A single constant concentration of 5-FAM-RORγt was incubated with increasing concentration of Cy5-18:1–18:1-PC in the presence of either recombinant WT Raftlin1 or Raftlin1^ΔLLNSL^. The fluorescence emission spectra of the mixtures were determined at an excitation wavelength of 470 nm. As the concentration of Cy5-18:1–18:1-PC was increased, the emission intensity of 5-FAM-RORγt at 560 nm gradually decreased in the presence of WT Raftlin1 but not with Raftlin1^ΔLLNSL^ (Supplementary Fig. 8d), indicating that 5-FAM-RORγt proteins were bound to Cy5-18:1–18:1-PC and strongly quenched by acceptor Cy5-18:1–18:1-PC in FRET assays. These results suggested that the interaction of RORγt and Raftlin1 is essential for the binding of phospholipid to RORγt.

To further confirm the role of phospholipids on the RORγt-Raftin1 mediated regulation of IL-17 expression in vivo, we adoptively transferred WT and Raftlin1^∆LLNSL^ Th17 cells cultured in the presence of the above-mentioned phospholipids into *Rag1*^*–/–*^ mice^26^. The *Rag1*^*–/–*^ mice that received WT Th17 cells cultured in the presence of phospholipids showed increased loss of body weight and higher fecal occult blood levels (Fig. 5d, e) compared to control WT Th17 cells. Colonoscopy analysis showed more inflammation and bleeding in the colon of *Rag1*^*–/–*^ mice that received WT Th17 cells cultured in the presence of phospholipids as compared to control WT Th17 cells (Fig. 5f). These mice also exhibited severe inflammation and increased shortening of the colon compared to mice that received control WT Th17 cells (Fig. 5g). Further, flow cytometry analysis showed an increased number of IL17^+^ and IL-17^+^IFN-γ^+^ cells in the lamina propria (Fig. 5h, i, Supplementary Fig. 9) of *Rag1*^*–/–*^ mice that received WT Th17 cells grown in the presence of phospholipids as compared to control WT Th17 cells. As expected, such an increase in IL-17^+^IFN-γ^+^ cells was not observed in *Rag1*^*–/–*^ mice that received Raftlin1^∆LLNSL^ Th17 cells. Similarly, the histologic analysis showed increased infiltration of inflammatory cells, more crypt damage, and higher disease scores in the *Rag1*^–/–^ mice receiving Th17 cells grown in the presence of phospholipid (Fig. 5j, k) but had no effect of phospholipids on *Rag1*^–/–^ mice receiving Raftlin1^∆LLNSL^ Th17 cells. Together, these findings indicate that Raftlin1 recruits phospholipids to RORγt and drives the pathogenicity of Th17 cells (Fig. 5l).

## Discussion

Th17 cells exhibit diverse functions ranging from host defense against microbial pathogens, maintenance of gut homeostasis, and a role in autoimmune diseases. The underlying mechanisms that regulate such diverse and opposing functions of Th17 cells remain an open question. Addressing this unresolved issue is critically important because Th17 targeting therapies have shown promising results in some but not all autoimmune diseases, even when Th17 cells are genetically linked to disease progression^29–33^. We have experimentally addressed this issue and have identified functional regulators of Th17 pathogenicity using a mass spectrometry and lipidomics-based approach.

In mouse models, Th17 cells in the gut are induced by commensal SFBs, which play an important role in barrier integrity and gut homeostasis^24,34–37^. The Th17 cells induced in response to SFBs do not cause epithelial cell damage and inflammatory infiltration of lamina propria^38^. Similar to SFB, the intestinal pathogen *C. rodentium* adheres to epithelium^39^, but unlike SFB, it causes epithelial cell death through effectors released via the type III secretory system, resulting in an inflammatory Th17 response^40^. Our results show that Raftlin1 is selectively upregulated in pathogenic Th17 cells induced in response to *C. rodentium* and directly binds to RORγt via its LLNSL motif; disruption of the RORγt-Raftlin1 complex significantly attenuates pathogenic Th17 cells. What triggers Raftlin1 expression following *C. rodentium* infection remains unknown. *C. rodentium* expresses a variety of pathogen-associated molecular patterns (PAMPs) and danger-associated molecular patterns (DAMPs)^41^. Previous studies have shown that membrane-bound transcription factors and coactivators respond rapidly to stresses from extracellular and intracellular stimuli^42^. It is possible that specific PAMPs or DAMPs of *C. rodentium* induce Raftlin1 expression. Also, *C. rodentium* activates multiple immune pathways, including IL-1β, Type 1 IFN, and IL-23^41^, which could modulate Raftlin1 expression. A systematic screening of the factors that induce Raftlin1 expression is essential to clearly understand the Raftlin1-RORγt pathway in the pathogenesis of Th17 cells.

Previous studies have shown that RORγt is sufficient to induce transcriptional activity (unlike other NRs) without exogenous agonists^3^. This suggests that RORγt is constitutively active and does not require a ligand. However, RORγt failed to induce transcription when ectopically expressed in *Drosophila melanogaster* cells grown in serum-free media. The transcriptional activity was restored by supplementing the culture with serum^12^. These findings suggested that endogenous lipid ligands may be required for RORγt transcriptional activity. Cholesterol and cholesterol derivatives are shown to restore RORγt activity when *Drosophila* cells are grown in a lipid-free chemically defined medium^12^. Cholesterol itself, as well as 20α-hydroxycholesterol (20 α-OHC), 22*R*-hydroxycholesterol (22*R*-OHC), and 25-hydroxycholesterol (25-OHC) induced RORγt transcriptional activity^12^. Similarly, another study showed that oxysterol, 7β,27-OHC, acts as a RORγt ligand^11^. By utilizing an unbiased (LC-MS-based) approach, we show here that phospholipids bind to RORγt and modulate the pathogenicity of Th17 cells in vivo. In line with our results, injection of lysophosphatidylcholine, a hydrolyzed form of phosphatidylcholine, enhanced Th17-mediated skin inflammation in the contact hypersensitivity model induced by 2,4-dinitrofluorobenzene (DNFB)^43^. How these phospholipids induce distinct transcriptional profiles in pathogenic Th17 cells remains to be investigated.

Increasing evidence shows that phospholipids and similar amphipathic molecules functionally regulate the activity of several NRs^44^. How do these mostly abundant phospholipids activate NRs? Small chemical changes in phospholipids (altering the number or position of unsaturated double bonds or the length of the acyl chains) affect their association with PPARα^45^. Similarly, the oxidation of phosphatidylcholine (oxPC) increases the activation of intracellular PPARα and PPARγ^46,47^. CD36, a plasma membrane scavenger receptor, enhances oxPC transport to specific intracellular compartments and facilitates oxPC activation of PPARγ target genes^46^. Additionally, it was shown that Phosphatidic acid (PA) generated by the action of Diacylglycerol (DAG) kinases on DAG activates SF1 and its target genes^48^. Interestingly, DAG binds to SF1 directly via the NR interaction LXXLL motif (similar to Raftlin1), suggesting a link between phospholipid metabolism and NR activation^48^. However, it remains an open question how NRs acquire these hydrophobic phospholipids from cellular membrane systems. While the phospholipid transfer proteins (PLTPs) are known to exist, it is unknown if they facilitate the exchange of phospholipids from the cellular membrane into NRs^44^. We demonstrate here that Raftlin1 facilitates the binding of phospholipids to RORγt. Our results show an association of Raftlin1 with RORγt in the nucleus, but how Raftlin1 translocates to the nucleus remains to be further investigated. Membrane proteins translocate and regulate transcriptional activity by multiple mechanisms (e.g., via proteolytic cleavage, depalmitoylation, and endocytosis followed by endoplasmic retrograde trafficking and nuclear translocation)^42^. A further detailed investigation is necessary to fully understand how Raftlin1 translocates to the nucleus of Th17 cells during an inflammatory response.

Raftlin1 was originally identified as a lipid raft protein involved in B cell receptor signaling^14^. *Rftn1*^–/–^ mice exhibit reduced T cell-dependent antibody production and less severe EAE compared to WT controls, whereas transgenic expression of Raftlin1 results in severe EAE^15^. Raftlin1 deficiency also results in reduced T cell receptor signaling^15^. We show that Raftlin1 binds to the AF2 domain of RORγt through its LLNSL motif. Disruption of this association does not affect TCR signaling or differentiation of Th1 and Th2 cells but markedly affects the generation of pathogenic Th17 cells. The AF2 domain of NRs within LBD is composed of the Helix 12, or the activation function helix AF-H, which has been shown to be conformationally dynamic, altering the orientation of AF2 to facilitate interaction with different coregulators^49^. The steroid receptor coactivators (SRCs) are shown to bind to the AF2 domain of RORγt and regulate thymocyte survival and IL-17 transcription^50^. The binding of SRC-1 displaces bound Foxp3 to RORγt, leading to the degradation of Foxp3 via a ubiquitin-proteasomal pathway and hence reversing the inhibitory action of Foxp3 on RORγt activity^51^. Therefore, it is possible that Th17 cells switch between SRCs and Raftlin1 in nonpathogenic and pathogenic Th17 cells. Crystallographic and Cryo-EM studies should provide more detailed mechanistic insights into how different coregulators modulate the RORγt function and pathogenicity of Th17 cells.

In conclusion, our studies indicate that Raftlin1 recruits distinct phospholipids to RORγt and drives the pathogenicity of Th17 cells. These results may provide a platform for therapeutic strategies to dampen Th17-mediated inflammation in various human diseases.

## Methods

### Mice

C57BL/6 (WT) mice and *Rag1*^*–/–*^ were purchased from Jackson Laboratory. All experiments were performed in accordance with the approved protocols by the Institutional Animal Care and Use Committee (IACUC) of UT Southwestern Medical Center (IACUC No.: 2019-102734, 2019-102735).

### Antibodies and Reagents

The following antibodies were used in this study: Anti-c-Myc (9E10, Santa Cruz biotechnology, sc-40, 1:1000), Anti-Flag (M2, Sigma Aldrich, F1804, 1:5000), Anti-RORγt (AFKJS-9, eBioscience, 14698882, 1:100), Anti-RORγt (O28-835, BD Bioscience, 562197, 1:500), Anti-GST (B14, Santa Cruz biotechnology, sc-138, 1:1000), Anti-β-actin (C4, Santa Cruz biotechnology, sc-47778, 1:1000), Anti-Phospho-Zap-70 (Tyr319)/Syk (Tyr352) (CST, 2701, 1:1000), Anti-Zap-70 (99F2, CST, 2705, 1:1000), Anti-Lck (3A5, Santa Cruz biotechnology, sc-433, 1:500), Anti-IκBα (CST, 9242, 1:2000), Anti-Raftlin (E11, Santa Cruz biotechnology, sc-514457, 1:1000), Anti-Raftlin-Alexa Fluor® 48 (E11, Santa Cruz biotechnology, sc-514457 AF488, 1:100), Anti-CD4-FITC (GK1.5, Biolegend, 100406, 1:200), Anti-CD4-PE (GK1.5, BD Bioscience, 553730, 1:200), Anti-CD4-APC (GK1.5, eBioscience, 17004181, 1:200), Anti-CD8-FITC (53-6.7, Biolegend, 100706, 1:200), Anti-CD25-FITC (PC61, Biolegend, 102006, 1:200), Anti-CD45RB-PE (16 A, BD Bioscience, 553101, 1:200), Anti-Foxp3-PE (FJK-16s, eBioscience, 12577380, 1:50), Anti-CD44-FITC (IM7, BD Bioscience, 553133, 1:100), Anti-CD62L-APC-efluorTM 780 (MEL-14, eBioscience, 47062182, 1:200), Anti-IL17-PerCP-Cy5.5 (TC11, Biolegend, 506920, 1:20), Anti- IL-22-PE (Poly5164, Biolegend, 516404, 1:20), Anti-IFN-γ-APC (XMG1.2, BD Bioscience, 554413, 1:50), Anti-mouse CD3 (17A2, Biolegend, 100223), Anti-mouse CD28 (37.51, BioXCell, BE0015), Anti-mouse IFN-γ (R4-6A2, BioXCell, BE0054), anti-IL4 (11B11, Biolegend, 504102), Clean-Blot™ IP detection reagent (HRP) (Themo Fisher Scientific, 21230, 1:200), Anti-mouse IgG for IP (HRP) (abcam, ab131368, 1:5000).

### Isolation of colonic lamina propria cells

Single-cell suspensions of colonic lamina propria cells were isolated from mouse colons (8–10 weeks of age) using Lamina propria dissociation kit (Miltenyi Biotec). For flow cytometry analysis, cells were stained with anti-CD4 antibody and intracellularly with anti-IL-17, anti-IL-22, anti-IFN-γ antibodies. For CD4^+^ T cell isolation, single-cell suspensions of colonic lamina propria cells were enriched for CD4^+^ T cells with mouse CD4^+^ T Cell Isolation kit (Miltenyi Biotec).

### Patients and tissue samples

Colonic mucosal biopsies were collected from non-IBD patients (healthy controls) undergoing colonoscopy for colorectal cancer screening/surveillance and from patients with UC undergoing diagnostic flexible sigmoidoscopy or colonoscopy. The study protocol was approved by the Institutional Review Board of UT Southwestern Medical Center (STU 112010-130). Written informed consent was obtained from all patients that were recruited. In healthy controls, biopsies were collected from the normal mucosa of the sigmoid colon. In UC patients, biopsies were collected from the actively inflamed mucosa of the sigmoid colon based on endoscopic appearances. Active inflammation was subsequently confirmed by histologic findings. A total of 3-6 pieces of mucosal biopsies were collected from each patient and were used for further applications.

### Generation of Knock-in mice by CRISPR-Cas9

Raftlin1 knock-in mice lacking LLNSL motif were generated using the CRISPR-Cas9 approach. To delete the LLNSL motif on Exon 8 of *Rftn1*, specific guide RNA sequences and homology-directed repair (HDR) templates were used. The top hit gRNA was selected from gRNA design tool CRISPOR. 20 mer guide sequences were synthesized by Synthego, USA, as a modified RNA sequence (GACUCGCUGGCGGCCUACGGA). For HDR, Alt-R^TM^ HDR donor oligos of 156 mer (5ʹ-TGAATCAATGGGAGGATAAACTCAAGTGGTGAGGAGGTGACCTTAGGTGATAGCAGTGCTCACGGTGGCCTTTCTCCACAGGGTACAGAGGTACAGACCGACTACATGCCCGCGGCCTACGGATGGCAGCTCACCTGTGTGCTGCCAACGCCTATA-3ʹ) were synthesized from IDT, USA. *Rftn1* RNAs, Cas9 protein, and HDR donor oligos were injected into the pro-nuclei of fertilized eggs. The pups were screened for the Raftlin1 knock-in region at exon 8 by PCR (Supplementary Fig. 3a). To explore the potential off-target loci, the gRNA used for the targeting was checked with CRISPOR. Fifteen off-target genes were found to match with gRNA (Supplementary Table 2). The specific primer pairs were designed to amplify the top nine predicted off-target regions and sorted by the cutting frequency determination (CFD) off-target score (Supplementary Tables 2–3). Off-target regions with very low CFD scores <0.02 are unlikely to be cleaved. The PCR amplified fragments were analyzed by Sanger sequencing and aligned with the mouse genome to determine the off-target effects of all the five loci (Supplementary Fig. 3b). The mice with no off-target effects were chosen for subsequent experiments.

### Molecular cloning and protein expression

The Myc-Raftlin1 plasmid was generated by cloning of PCR amplified *Rftn1* from mouse cDNA into pcDNA3.1(+) C-Myc vector. The mutant Raftlin1^ΔLLNSL^ plasmid was generated by site-directed mutagenesis (Q5® Site-Directed Mutagenesis Kit, NEB) using Myc-Raftlin1 as a template and deletion of LLNSL motif was confirmed by Sanger sequencing. GST expression clones of Raftlin1 and Raftlin1^ΔLLNSL^ were constructed by first subcloning into Gateway™ pENTR™ 3C entry vector (Invitrogen). Then LR recombined using Gateway™ LR Clonase™ II Enzyme mix (ThermoFisher Scientific, USA) to pGEX-6P1-DEST vector (Addgene plasmid # 119749) containing GST tag at N-terminal. The sequences of all clones were verified by Sanger sequencing. Further, GST-Raftlin1 and GST- Raftlin1^ΔLLNSL^ proteins were individually expressed in Rosetta™(DE3)pLysS competent cells (Novagen, USA). Cleared lysates were prepared, and the soluble fusion proteins were purified on glutathione Sepharose 4B GST-tagged protein purification resin (GE Healthcare Life Sciences, USA), and the purity of proteins was checked by Coomassie blue staining on SDS-PAGE.

### *C. rodentium* infection and quantification

For *C. rodentium* infection, a single colony of strain DBS100 (ATCC 51459; American Type Culture Collection) was transferred to Luria-Bertani (LB) broth and grown to log phase, followed by centrifugation and resuspension in PBS. Mice (8–10 weeks of age) were orally gavaged with 200μl of PBS containing 2 × 10^9 ^*C. rodentium* CFU. To determine the bacterial load, feces were collected, weighed, and homogenized in sterile PBS, and serial dilutions were plated onto MacConkey agar for measurement of colony-forming units (CFU) and confirmed by PCR using *C. rodentium* gene-specific primers.

### Segmented Filamentous Bacteria (SFB) colonization and quantification

Fecal pellets from SFB-monocolonized mice^25^ were vortexed for 10 min in PBS. The fecal suspension was centrifuged at 1000 × *g* for 2 min, and 200 μl of the supernatant was used to gavage mice. After 2–3 weeks, fecal pellets were collected, and DNA was purified using Qiagen stool DNA purification kit. Next, 1μg of DNA from each sample was used for real-time PCR with SFB 16 S rRNA gene sequence-specific primers using SYBR green kit. PCR reactions were analyzed using standard curves generated with template controls designed for the above primer set that were run along with the experimental samples.

### In vitro T cell differentiation

For in vitro experiments, naïve CD4^+^ T cells were isolated from the spleen of WT and Raftlin1^ΔLLNSL^ mice (8–10 weeks of age) using a naïve CD4^+^ T cell isolation kit (Miltenyi Biotec, USA). The cells were cultured in a 24-well cell culture plate with plate-bound anti-CD3 (1 µg/ml) and soluble anti-CD28 (2 µg/ml) antibodies. For Th1 differentiation, rIL-12 (2 ng/ml) and anti-IL-4 (10 µg/ml) were added in the culture media. For Th2 differentiation, rIL-4 (10 ng/ml) and anti-IFN-γ (10 µg/ml) were added. For Th17 differentiation, TGF-β (5 ng/ml), IL-6 (10 ng/ml), IL-23 (50 ng/ml), anti-IL-4 (10 μg/ml), and anti-IFN-γ (10 μg/ml) were added and cultured for 5 days^52,53^. Where indicated, cultures were supplemented with 20 µM phospholipids.

### Adoptive transfer of colitogenic cells in *Rag1*^*−/−*^ mice

Naïve CD4^+^ T cells were isolated from the spleen of wild-type (WT) and Raftlin1^ΔLLNSL^ mice using CD4^+^ T cell isolation kit (Miltenyi Biotec, USA). Cells were then stained with antibodies against CD45RB, CD25, and CD4, and then FACS sorted for CD4^+^CD25^–^CD45RB^hi^ T cell populations. The FACS-sorted CD4^+^CD25^–^CD45RB^hi^ cells (5 × 10^5^ cells/mice) were injected intraperitoneally into 8-weeks-old *Rag1*^*–/–*^ mice, and the mice were monitored for body weight, diarrhea score, and fecal occult blood (FOB) score up to 7 weeks. For Th17 cell-induced colitis model, naïve CD4^+^ T cells were isolated from the spleen of WT and Raftlin1^ΔLLNSL^ mice using a naïve CD4^+^ T cell isolation kit. The purified cells were differentiated into Th17 cells in the presence of plate-bound anti-CD3 (1 μg/ml) antibody. The cells were cultured under Th17-inducing conditions for 5 days as discussed above, in the presence of phospholipids. *Rag1*^−/−^ mice were injected intra-peritoneally with Th17 cells (5 × 10^5^ cells/mice) and monitored for disease severity for up to 7 weeks.

### GM1 staining and lipid raft analysis in T cells

CD4^+^ T cells were isolated from the spleen of WT and Raftlin1^ΔLLNSL^ mice and activated with anti -CD3 (1 μg/ml) and anti-CD28 (2 μg/ml) immobilized on 24 well plates for 48 h^54^. The cells were fixed with 0.75% paraformaldehyde and permeabilized with a buffer containing 0.05% (w/v) saponin in PBS and stained with cholera toxin B (CT-B)-PE for 1 h. Further, cells were stained with Hoechst 22258 and transferred to slides. The images were acquired using Keyence microscopy.

### Proximity ligation assay

Proximity ligation assays (PLA) were performed using the Duolink® PLA Fluorescence protocol (Sigma-Aldrich). In brief, CD4^+^ T cells were isolated from *C. rodentium* infected and uninfected mice. The cells were fixed with 4% paraformaldehyde for 20 min at 4 °C and permeabilized with 0.2% saponin, followed by blocking in 5% BSA. After that, the cells were incubated with rabbit primary antibody against Raftlin1 and mouse primary antibody against RORγt, and secondary antibodies with PLA probes were added for hybridization. Duolink® PLA probes in the absence of primary antibodies were used as control. The samples were incubated with ligase and polymerase for the ligation and rolling circle amplification. After ligation and amplification steps, the slides were mounted with a cover slip using Mounting Medium with DAPI and analyzed on a confocal microscope (Zeiss LSM880). Each individual red dot represents a high concentration of fluorescence as a result of the probe proximity and signifies a direct interaction between two proteins.

### Fluorescence resonance energy transfer assay

Fluorescence resonance energy transfer (FRET) assays were conducted using donor 5-FAM (5-Carboxyfluorescein) labeled RORγt protein and acceptor Cyanine 5 (Cy5) labeled 1, 2-dioleoyl-sn-glycero-3-phosphatidylcholine (18:1–18:1-PC) (Avanti Polar lipids, USA) FRET pair. The purified RORγt protein was fluorescently labeled with 5-FAM by N-hydroxysuccinimide (NHS) ester-mediated derivatization^55^. Purified RORγt protein was dialyzed against the reaction buffer containing 0.1 M Sodium phosphate buffer, pH 7.0, 75 mM Potassium acetate, pH 7.4, and 2 mM DTT, and incubated in the dark with 5-FAM at a 20:1 molar ratio for the labeling. The labeled RORγt was further dialyzed against storage buffer containing 20 mM HEPES-KOH, pH 7.4, 100 mM Potassium acetate, pH 7.4, 2 mM DTT, and 10% glycerol, and loaded onto Amicon protein concentrator (Millipore Sigma, USA) to concentrate the protein and remove the free fluorophore. 5-FAM labeled RORγt and Cy5 labeled phospholipid were incubated with GST-Raftlin1 or GST-Raftlin1^ΔLLNSL^ in a reaction buffer containing 50 mM Tris, pH 7.5, 100 mM NaCl and 10 mM CaCl_2_. Fluorescence emission spectra at 560 nm were obtained with the increasing concentration of Cy5 labeled phospholipid (0–10^4^ nM) in the reaction mixture using BioTek SYNERGY H1 microplate reader. The fluorescence measurement was performed in 96 well microplates with the excitation wavelength at 470 nm.

### Flow cytometry and cell sorting

Single-cell suspensions were prepared from the spleen, mLN, thymus, payers patch (PP), and colon, and stained with viability dye (live/dead aqua). The cells were further stained with antibodies against CD4, CD8, CD25, CD44, and CD62L, fixed and permeabilized in BD Fix/Perm buffer, and stained intracellularly with anti-IL-17, anti -IL-22, anti -IFN-γ antibodies. For intracellular staining, single-cell suspensions were first stimulated with 50 ng/ml phorbol-12-myristate-13-acetate (PMA), 1 μg/ml ionomycin in the presence of GolgiStop^TM^ (BD Biosciences) for 4 h before staining. For CD4^+^CD25^–^CD45RB^hi^ T cell sorting, single-cell suspensions from the spleen were enriched for CD4^+^ T cells with Mouse CD4^+^ T Cell Isolation kit according to the manufacturer’s instructions. Cells were stained with antibodies against CD4, CD25, and CD45RB and sorted as live CD4^+^CD25^–^CD45RB^hi^ cells for adoptive transfer experiments. Samples were run on a BD FACSCanto^TM,^ and analysis was performed with FlowJo v10 software.

### Experimental encephalitis (EAE) model

WT and Raftlin1^ΔLLNSL^ mice, 8–10 weeks of age, were immunized subcutaneously on day 0 with 100 μg MOG (35–55) peptide. Pertussis toxin (Enzo Life Sciences) in 100 μl (200 ng) saline was injected subcutaneously twice (once each on days 0 and 1). Disease severity was assigned based on the following scale: 0, no disease; 0.5, stiff tail; 1, limp tail; 1.5, limp tail with the inability to right; 2, paralysis of one limb; 2.5, paralysis of one limb and weakness of another limb; 3, complete paralysis of both hind limbs; 4 moribund; 5, death^18^.

### CD4^+^ T cell isolation from biopsy samples

Three to six biopsy specimens were cut into small pieces and incubated in a digestion buffer containing Collagenase (0.5 mg/ml), DNase I (100 μg/ml), and 10 mM HEPES in RPMI with 2% heat-inactivated FBS^56^. Briefly, three rounds of digestion were applied on 3-6 pieces of biopsies in 3 ml of digestion buffer, mechanical disruption using a blunt needle, filtration and wash by passing through a 70 μm cell-strainer. After washing, three aliquots of cells were then combined in MACS buffer (PBS, pH 7.2, 0.5% BSA, 2 mM EDTA) for T cell isolation using a CD4^+^ T cell isolation kit (Miltenyi Biotec).

### Real-time PCR analysis

Total RNA was prepared using the RNeasy Mini kit or RNeasy Micro Kit (Qiagen) followed by cDNA synthesis using the Verso cDNA Synthesis Kit (ThermoFisher Scientific). Quantitative real-time PCR was performed on a Mastercycler Realplex2 (Eppendorf, Hamburg, Germany) using lightCycler 480 SYBR-Green Master Mix. All reactions were performed in triplicate. The expression of individual genes was normalized to the expression of β-Actin. The following cycling parameters were used: 95 °C for 2 min, followed by 40 cycles of 95 °C for 15 s, an annealing temperature of 55 °C for 15 s, and amplification at 72 °C for 20 s. The primer sequences for the genes are as follows:

mIL-17A forward primer:5ʹ-TTTAACTCCCTTGGCGCAAAA-3ʹ

mIL-17A reverse primer:5ʹ-CTTTCCCTCCGCATTGACAC-3ʹ

mRaftlin1 forward primer:5ʹ-GAAGGCTGAGCTTCACGACGAAG-3ʹ

mRaftlin1 reverse primer:5ʹ-CGGCCTCCTGCACCTTAATGAAC-3ʹ

mRaftlin2 forward primer:5ʹ-TGTGAGGCACAAGCAAACGAC-3ʹ

mRaftlin2 reverse primer:5ʹ-GCATAGATGGCAAGTAGCACTGG-3ʹ

mβ-Actin forward primer:5ʹ-GAAATCGTGCGTGACATCAAAG-3ʹ

mβ-Actin reverse primer:5ʹ-TGTAGTTTCATGGATGCCACAG-3ʹ

hIL-17A forward primer:5ʹ-CATCCATAACCGGAATACCAATA-3ʹ

hIL-17A reverse primer:5ʹ-TAGTCCACGTTCCCATCAGC-3ʹ

hRaftlin1 forward primer:5ʹ-CTGGATGGACCGGAGAGCAAC -3ʹ

hRaftlin1 reverse primer:5ʹ-GTATTGCCGGCACTTCTGATGGC-3ʹ

hβ-Actin forward primer:5ʹ-CTACGTCGCCCTGGACTTCGAGC-3ʹ

hβ-Actin reverse primer:5ʹ-GATGGAGCCGCCGATCCACACGG-3ʹ

mIL4 forward primer:5ʹ-GGTCTCAACCCCCAGCTAG-3ʹ

mIL4 reverse primer:5ʹ-GCCGATGATCTCTCTCAAGT-3ʹ

mIL2 forward primer:5ʹ-GTGCTCCTTGTCAACAGCG-3ʹ

mIL2 reverse primer:5ʹ-GGGGAGTTTCAGGTTCCTGTA-3ʹ

mIL23 forward primer:5ʹ-GCTCGGATTTGGTATAAAGG-3ʹ

mIL23 reverse primer:5ʹ-ACTTGGTATCTATGTAGGTAGG-3ʹ

mIFNγ forward primer:5ʹ-GAACTGGCAAAAGGATGGTGA-3ʹ

mIFNγ reverse primer:5ʹ-TGTGGGTTGTTGACCTCAAAC-3ʹ

16s-SFB forward primer:5ʹ-GACGCTGAGGCATGAGAGCAT-3ʹ

16s-SFB reverse primer:5ʹ-GACGGCACGGATTGTTATTCA-3ʹ

espB -forward primer:5ʹ-GCTTCTGCGAAGTCTGTCAA-3ʹ

espB- reverse primer:5ʹ-CAGTAAAGCGACTTAACAGATT-3ʹ

### Chromatin immunoprecipitation (ChIP) assay

The cells were cross-linked with 1% (v/v) methanol-free formaldehyde for 10 min and processed according to the protocol described in the Chromatin Immunoprecipitation Assay Kit (Millipore, USA). Antibody chromatin complexes were pulled down using protein A/G beads, washed, and then eluted. After cross-link reversal and proteinase K treatment, immunoprecipitated DNA was purified using the ChIP DNA Clean kit (Zymo Research, Irvine, CA, USA), and PCR was carried out with appropriate primers using an equal amount of precipitated DNA. In re-ChIP assay, the first ChIP was performed with either anti-RORγt or anti-Raftlin1 antibodies. The eluant of each immunocomplex was further diluted 10-fold with ChIP dilution buffer and then subjected to immunoprecipitation with second antibodies, either anti-Raftlin1 or anti-RORγt, respectively. The primer sequences for PCR are as follows:

mIL-17 promoter forward primer:5ʹ-GACAGATGTTGCCCGTCATA-3ʹ

mIL-17 promoter reverse primer:5ʹ-CAACAAGCGCCTTGTACATTAG-3ʹ

### Luciferase assay

Jurkat T-cells (Clone E6-1, ATCC, TIB-152™) were transfected with Flag-RORγt, mIL-17 promoter plasmid, and either wild-type Raftlin1 or mutant Raftlin1^ΔLLNSL^ using the Amaxa Cell Line Nucleofector kit (Lonza biosciences). After 24 h of transfection, cells were stimulated with PMA (50 ng/ml) and ionomycin (1 μg/ml) for 4 h. Lysates were prepared using the Dual-Luciferase Reporter Assay System kit (Promega), and luminescence was measured by Synergy H1 Multi-mode Microplate Reader (Bio TeK).

### Immunoblot analysis and immunoprecipitation

Cells were lysed in the NP-40 lysis buffer (50 mM Tris-HCl, pH 7.4, 150 mM NaCl, 1% NP-40, supplemented with cocktail protease inhibitor). Plasmids were transfected in HEK293T cells (ATCC, CRL-3216™) using lipofectamine (Invitrogen). Protein estimations were done using the Pierce BCA protein assay kit. Protein samples were resolved on SDS-PAGE and were transferred to the polyvinylidene difluoride (PVDF) membrane by a wet blot system. Post-transfer, the membrane was transferred to a blocking buffer (phosphate-buffered saline, 5% skimmed milk, and 0.1% Tween-20) for 1 h at room temperature. After incubation, the blot was washed 3 times (10 min each) with washing buffer (Tris-buffered saline and 0.1% Tween-20). Subsequently, the membrane was incubated overnight at 4 °C with primary antibodies diluted in blocking buffer, followed by washing 3 times (10 min each) with washing buffer. The membrane was then incubated with secondary antibodies conjugated to poly-horseradish peroxidase (HRP) for another 1 h at room temperature. After subsequent washing, a blot was developed with ECL western blotting detection reagents. For immunoprecipitation, cells were harvested and lysed in NP-40 buffer at 4 °C for 20 min. After centrifugation, the supernatant was transferred to fresh tubes. Approximately 10% of the whole-cell lysate was used as input. Whole-cell lysates were precleared with 20 μl of Protein A/G plus agarose beads for 1 h at 4 °C. Lysates were then incubated with 1μg of the desired antibody overnight at 4 °C followed by 1 h incubation at 4 °C with 25 μl of Protein A/G beads. The immunocomplexes were washed 5 times with NP-40 buffer, denatured using 4X Laemmli buffer, separated by SDS-PAGE, transferred to PVDF membranes, and analyzed by immunoblotting. Clean-Blot^TM^ IP detection reagent (HRP) was used as a secondary antibody in all immunoprecipitation assays.

### Protein identification by liquid chromatography-tandem MS

Cell lysates were prepared from CD4^+^ T cells isolated from lamina propria cells of *C. rodentium* infected WT mice and incubated with anti-RORγt antibody or IgG control and immunoprecipitated using protein A/G-agarose beads. The immunoprecipitated proteins were digested with trypsin, followed by protein identification using liquid chromatography-tandem MS^57^. Briefly, tryptic peptides were resolved on a nano-liquid chromatography column (Magic AQ C18; Michrom Bioresources, Auburn, CA, USA) and introduced into an Orbitrap mass spectrometer (Thermo Scientific, Waltham, MA, USA). The Orbitrap was set to collect a high-resolution MS1 (FWHM 30,000@400 m/z), followed by the data-dependent collision-induced dissociation spectra on the “top 9” ions in the linear ion trap. Spectra were searched against a *Mus musculus* protein database (UniProt release v2016-4-13) using the X!Tandem/TPP software suite^57^. Proteins identified with a Protein Prophet probability ≥0.9 and a false discovery rate <2% were considered for further analysis.

### Structure modeling and docking of the RORγt-Raftlin1 complex

The crystal structure of the ligand binding domain from RORγt (PDB: 5X8U) and the modeled structure of Raftlin1 by AlphaFold2^58^ were used as inputs for the program HADDOCK2.4^59^ to generate the model of the complex between the two proteins. The PDB of the top solution with the highest score from HADDOCK2.4 was used for the study.

### Colonoscopy examination

A high-resolution murine video endoscopic system was employed to gauge the severity of the colitis. Mice were sedated with Ketamine/Xylazine, and endoscopy was carried out using a mini-endoscope from Karl-Storz to monitor colon inflammation in vivo. According to the mouse endoscopic index of colitis severity (MEICS), colitis scores were graded. In short, perianal findings, wall transparency, intestinal hemorrhage, and localized lesion were used to determine MEICS. Each of these four different inflammation parameters was given a score from 0 to 3, giving rise to a total MEICS that ranged from 0 to 12^60^.

### Immunoprecipitation (IP) and lipidomics

For endogenous IP, mLN cells were isolated from *C. rodentium* infected WT mice. Approximately 5 × 10^9^ cells/IP at a concentration of 1 × 10^9^ cells/ml were lysed in detergent-free buffer (20 mM Hepes pH 7.4, 1.5 mM MgCl_2_, 0.2 mM EDTA, 25% Glycerol, 420 mM KCl, 0.1 mM PMSF, 1 mM DTT, and protease inhibitor cocktail) and incubated on ice for 15-20 min. After incubation, cells were homogenized 6-7 times with a dounce homogenizer and spun at 15000 × *g* for 15 min. The supernatants were processed for immunoprecipitation^12^. The immunoprecipitation samples were incubated in a rotator shaker overnight at 4 °C, and protein A/G beads were added for 1 h. After incubation, beads were washed 4 times with 50 mM Tris HCl pH 7.4, 100 mM NaCl plus protease inhibitor cocktail, and proceeded for lipidomics.

Lipid species were analyzed using multidimensional mass spectrometry-based shotgun lipidomic analysis^61^. In brief, each immunoprecipitated sample homogenate containing 0.08 mg of protein (determined with a Pierce BCA assay) was accurately transferred to a disposable glass culture test tube. A pre-mixture of internal lipid standards (IS) was added prior to conducting lipid extraction for quantification of the targeted lipid species. Lipid extraction was performed using a modified Bligh and Dyer procedure, and each lipid extract was reconstituted in chloroform: methanol (1:1, v:v) at a volume of 100μl.

For shotgun lipidomics, lipid extract was further diluted to a final concentration of ~500 fmol total lipids per μl. Mass spectrometric analysis was performed on a triple quadrupole mass spectrometer (TSQ Altis, Thermo Fisher Scientific, San Jose, CA) and a Q Exactive mass spectrometer (Thermo Scientific, San Jose, CA), both of which were equipped with an automated nanospray device (TriVersa NanoMate, Advion Bioscience Ltd., Ithaca, NY)^62^. Identification and quantification of lipid species were performed using an automated software program. Data processing (e.g., ion peak selection, baseline correction, data transfer, peak intensity comparison, and quantitation) was performed^61^. The results were normalized to the protein content (nmol lipid/mg protein or pmol lipid/mg protein).

### Statistics

The number of mice, experiments, and statistical tests are shown for each figure in the figure legend. The data were analyzed with GraphPad Prism 9 software to determine statistical significance using a two-tailed unpaired Student’s *t* test. The data are expressed as mean ± SD. A *p* value  <  0.05 was considered significant. *p < 0.05; **p < 0.01; and ***p < 0.001.

### Reporting summary

Further information on research design is available in the Nature Portfolio Reporting Summary linked to this article.

### Supplementary information

Supplementary Information

Reporting Summary

### Source data

Source Data

## Data Availability

The authors declare that all data supporting the findings of this study are available within the article and its supplementary file or from the corresponding author upon reasonable request. Source data are provided with this paper.
