# Supplementary material for: RORγt-Raftlin1 complex regulates the pathogenicity of Th17 cells and colonic inflammation
Source: Nat Commun. 2023 Aug 17;14:4972. doi: 10.1038/s41467-023-40622-1 (PMC10435467; doi:10.1038/s41467-023-40622-1)

Supplementary Information

RORγt-Raftlin1 complex regulates the pathogenicity of Th17 cells and colonic inflammation.

Amir Kumar Singh[1,2,3], Ritesh Kumar[1,2,3], Jianyi Yin[1], John F. Brooks[2], Mahesh Kathania[1,2,3], Sandip Mukherjee[1,2,3], Jitendra Kumar[1,2,3], Kevin P. Conlon[4], Venkatesha Basrur[4], Zhe Chen[5], Xianlin Han[6], Lora V. Hooper[2,7], Ezra Burstein[1], and K Venuprasad[1,2,3]

Contents

Supplementary Figures 1-9

Supplementary tables 1-3

Uncropped western blots and PCR agarose gels of Supplementary figures

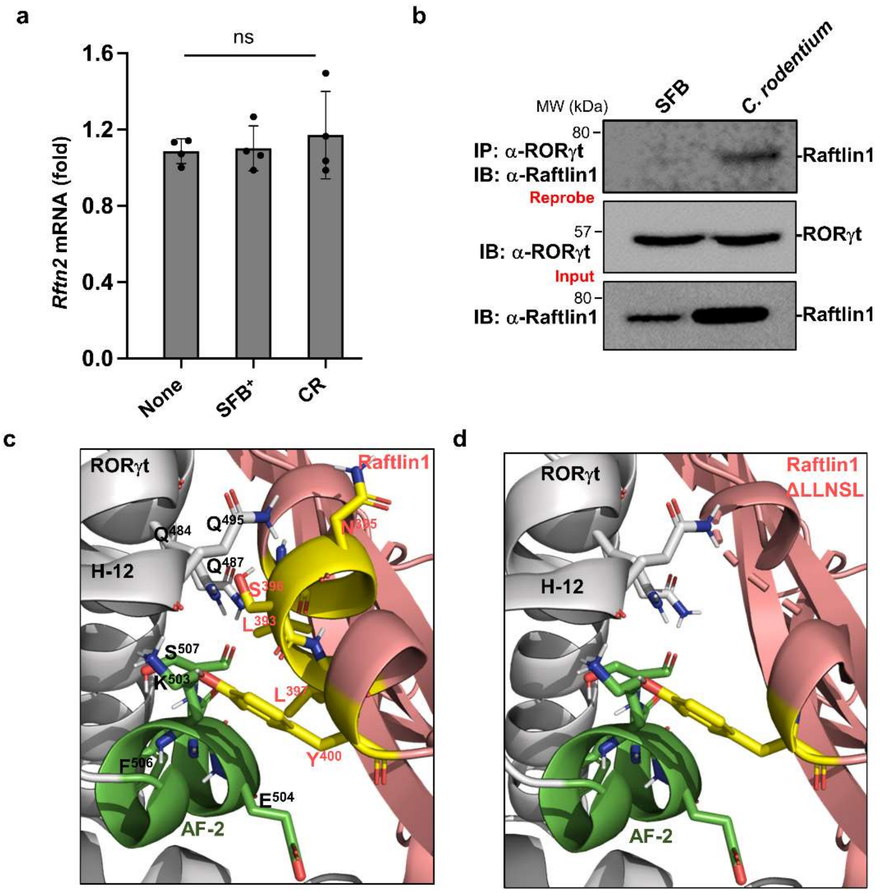

Supplementary Fig. 1: RORγt interacts with Raftlin1. **a**, Expression of *Rftn2* in CD4$^+$ T cells isolated from colonic lamina propria of *C. rodentium* infected, SFB colonized, and uninfected WT mice; n=4 mice per group, p=0.51. **b**, Immunoassay in the lysates of CD4$^+$ T cells isolated from colonic lamina propria of *C. rodentium* infected and SFB colonized WT mice were subjected to immunoprecipitation with anti-RORγt antibody and immunoblot analysis with anti-Raftlin1 or anti-RORγt antibody. Immunoblots are from one experiment representative of three independent experiments with similar results. **c**, The LBD domain of RORγt (PDB: 5X8U) and Raftlin1 (Alphafold2 predicted structure) are docked using HADDOCK2.4. The docked interface is shown as a cartoon diagram. The LLNSL motif of Raftlin1 makes non-covalent interaction with the AF2 domain and α-helix 12 of RORγt. The amino acids involved in non-covalent interactions are shown in the stick model. RORγt Helix-12 is shown in color grey, AF2 domain in color green, Raftlin1 in color salmon, and LLNSL motif of Raftlin1 in color yellow. **d**,

the protein-protein interface between RORγt and Raftlin1 is disrupted after deletion of the LLNSL motif in Raftlin1. Statistical significance (a) was determined by unpaired Student's t-test (two-tailed) with $p < 0.05$ considered statistically significant. ns- not significant, error bars are mean±SD. Source data are provided as a Source data file.

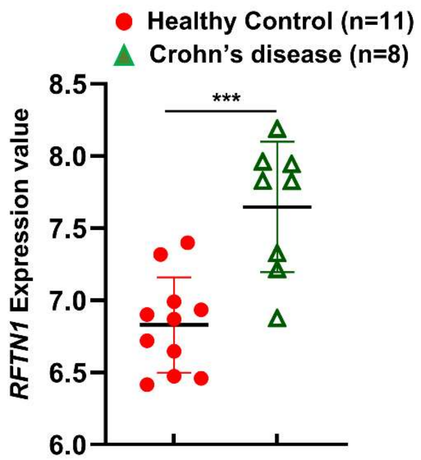

Supplementary Fig. 2: Raftlin1 expression in Crohn's disease patients. GEO dataset (GSE59071) was analyzed, and the normalized expression value of *RFTN1* is shown (n=11 healthy control and n=8 Crohn's disease patient's samples, p=0.0003). Statistical significance was determined by unpaired Student's t-test (two-tailed) with $p < 0.05$ considered statistically significant. ***$p < 0.001$, error bars are mean$\pm$SD. Source data are provided as a Source data file.

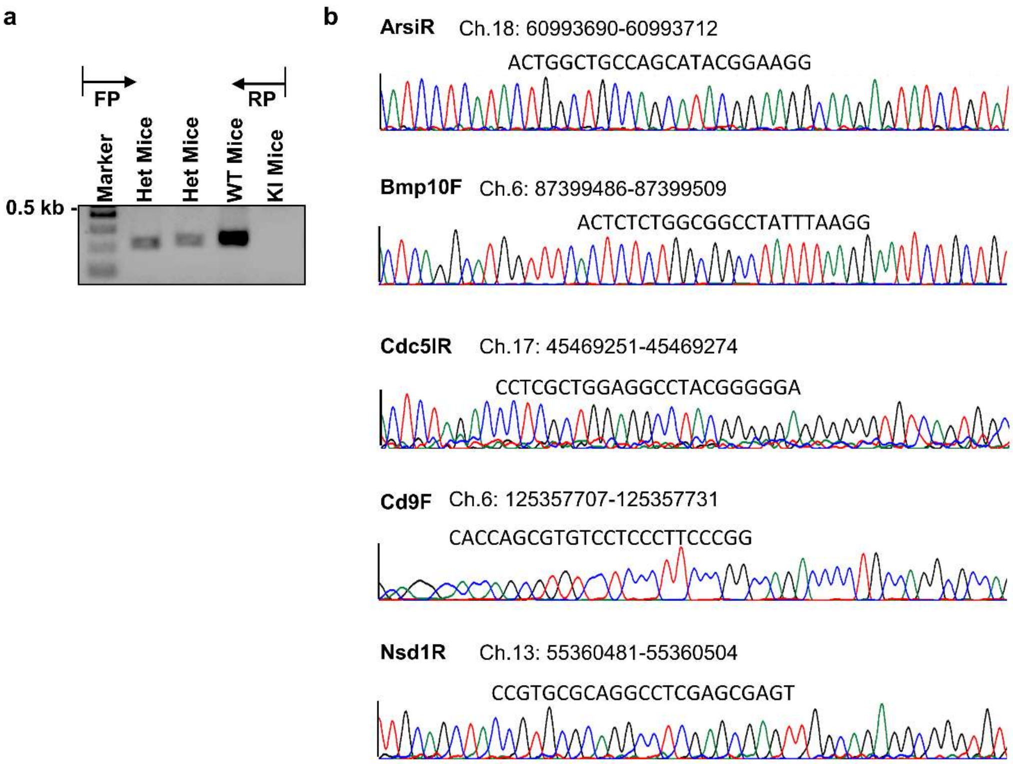

Supplementary Fig. 3: Genotyping and off-target gRNA analysis in the Raftlin1$^{\Delta LLNSL}$ mice. **a**, Genotyping of Raftlin1$^{\Delta LLNSL}$ mice by PCR. **b**, Representative Sanger sequencing chromatograms to check the potential off-target sites of gRNA in Raftlin1$^{\Delta LLNSL}$ mice. No off-target cleavage sites were detected.

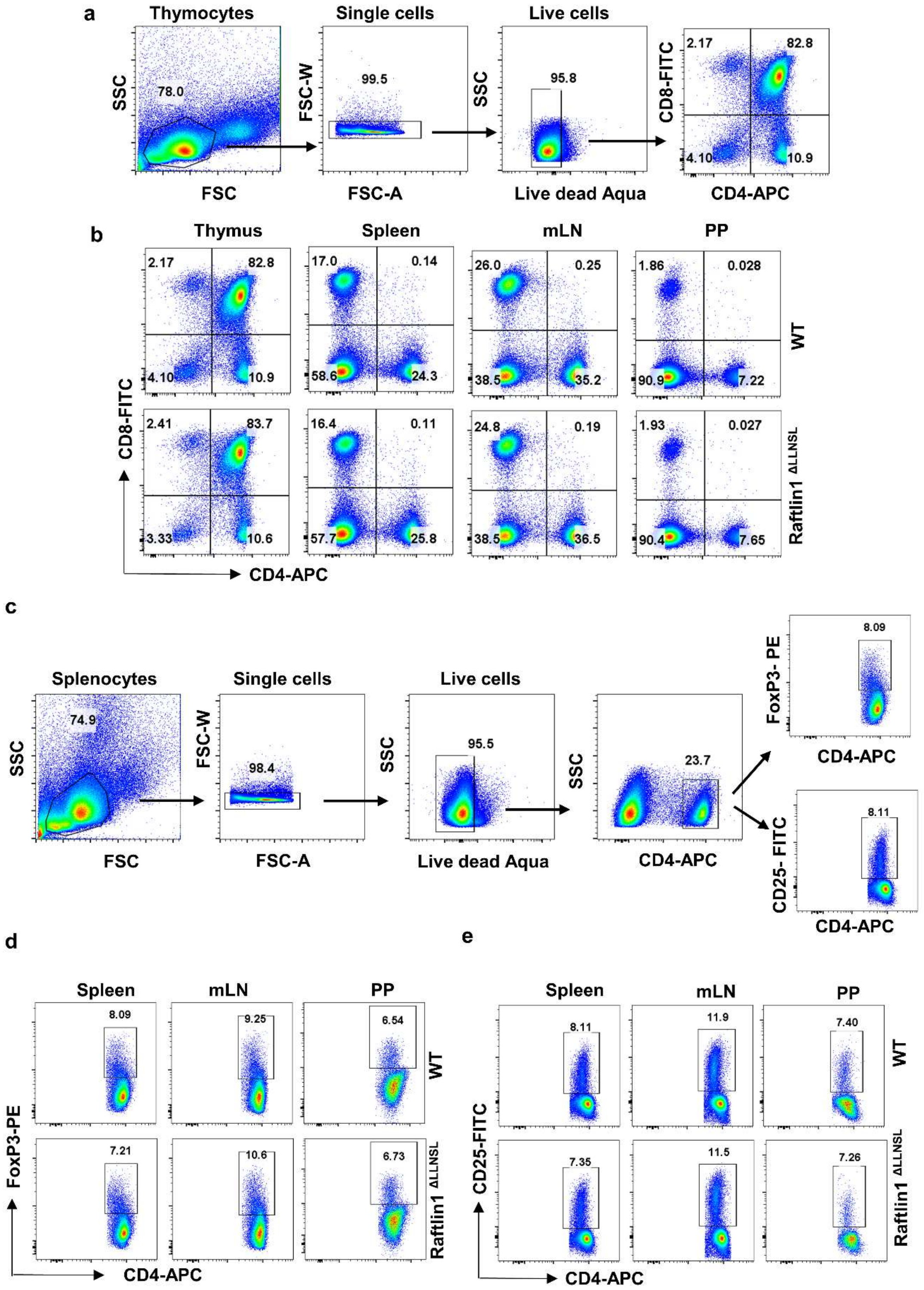
a
Thymocytes
Single cells
Live cells
SSC
78.0
FSC
FSC-W
99.5
FSC-A
95.8
Live dead Aqua
CD8-FITC
2.17
82.8
4.10
10.9
CD4-APC
b
Thymus
Spleen
mLN
PP
2.17
82.8
4.10
10.9
17.0
0.14
58.6
24.3
26.0
0.25
38.5
35.2
1.86
0.028
90.9
7.22
WT
2.41
83.7
3.33
10.6
16.4
0.11
57.7
25.8
24.8
0.19
38.5
36.5
1.93
0.027
90.4
7.65
Raftlin1 ΔLLNSL
CD8-FITC
CD4-APC
c
Splenocytes
74.9
SSC
FSC
Single cells
FSC-W
98.4
FSC-A
Live cells
SSC
95.5
Live dead Aqua
SSC
23.7
CD4-APC
FoxP3- PE
8.09
CD4-APC
CD25- FITC
8.11
CD4-APC
d
Spleen
mLN
PP
8.09
9.25
6.54
WT
7.21
10.6
6.73
Raftlin1 ΔLLNSL
FoxP3-PE
CD4-APC
e
Spleen
mLN
PP
8.11
11.9
7.40
WT
7.35
11.5
7.26
Raftlin1 ΔLLNSL
CD25-FITC
CD4-APC

f

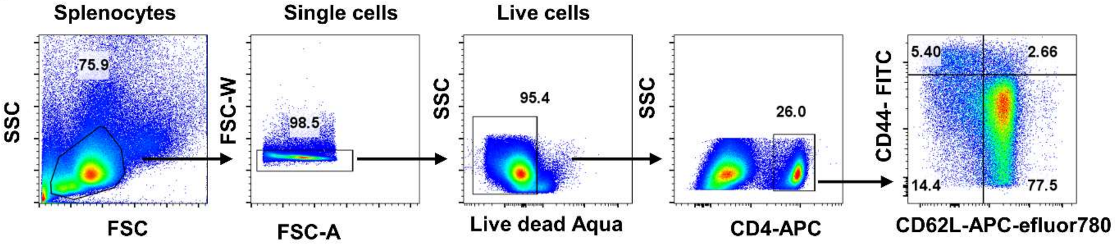

g

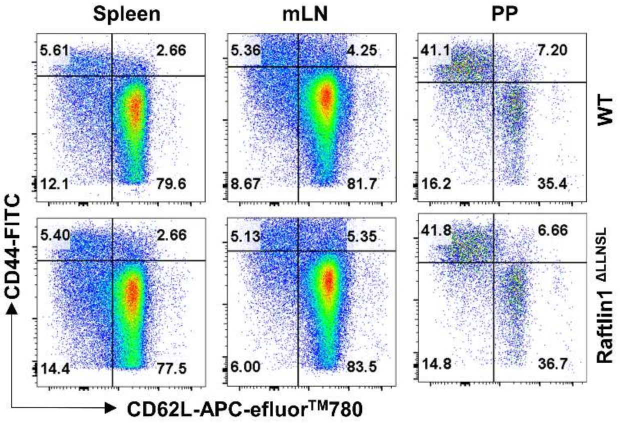

Supplementary Fig. 4: Flow cytometric analysis of T cells in the spleen, mesenteric lymph node (mLN), Peyer's patches (PP), and thymus of WT and Raftlin1$^{\Delta LLNSL}$ mice. a, Gating strategy of cells from thymus of WT and Raftlin1$^{\Delta LLNSL}$ mice stained with Live-Dead Aqua, anti-CD4, and anti-CD8 antibodies. **b**, Representative images of cells from thymus, spleen, mLN, and PP of WT and Raftlin1$^{\Delta LLNSL}$ mice were stained with antibodies against CD4 and CD8 and analyzed by flow cytometry. **c**, Gating strategy of cells from the spleen of WT and Raftlin1$^{\Delta LLNSL}$ mice stained with Live-Dead Aqua, anti-CD4, anti-FoxP3, and anti-CD25 antibodies. **d-e**, Representative image of cells from spleen, mLN, and PP of WT and Raftlin1$^{\Delta LLNSL}$ mice were stained with antibodies against CD4, FoxP3 and CD25, and analyzed by flow cytometry. **f**, Gating strategy of cells from the spleen of WT and Raftlin1$^{\Delta LLNSL}$ mice stained with Live-Dead Aqua, anti-CD4, anti-CD44, and anti-CD62L antibodies. **g**, Representative image of cells from spleen, mLN, and PP of WT and Raftlin1$^{\Delta LLNSL}$ mice stained with antibodies against CD4, CD44, and CD62L, and analyzed by flow cytometry. b, d, e, g n=5 mice per group.

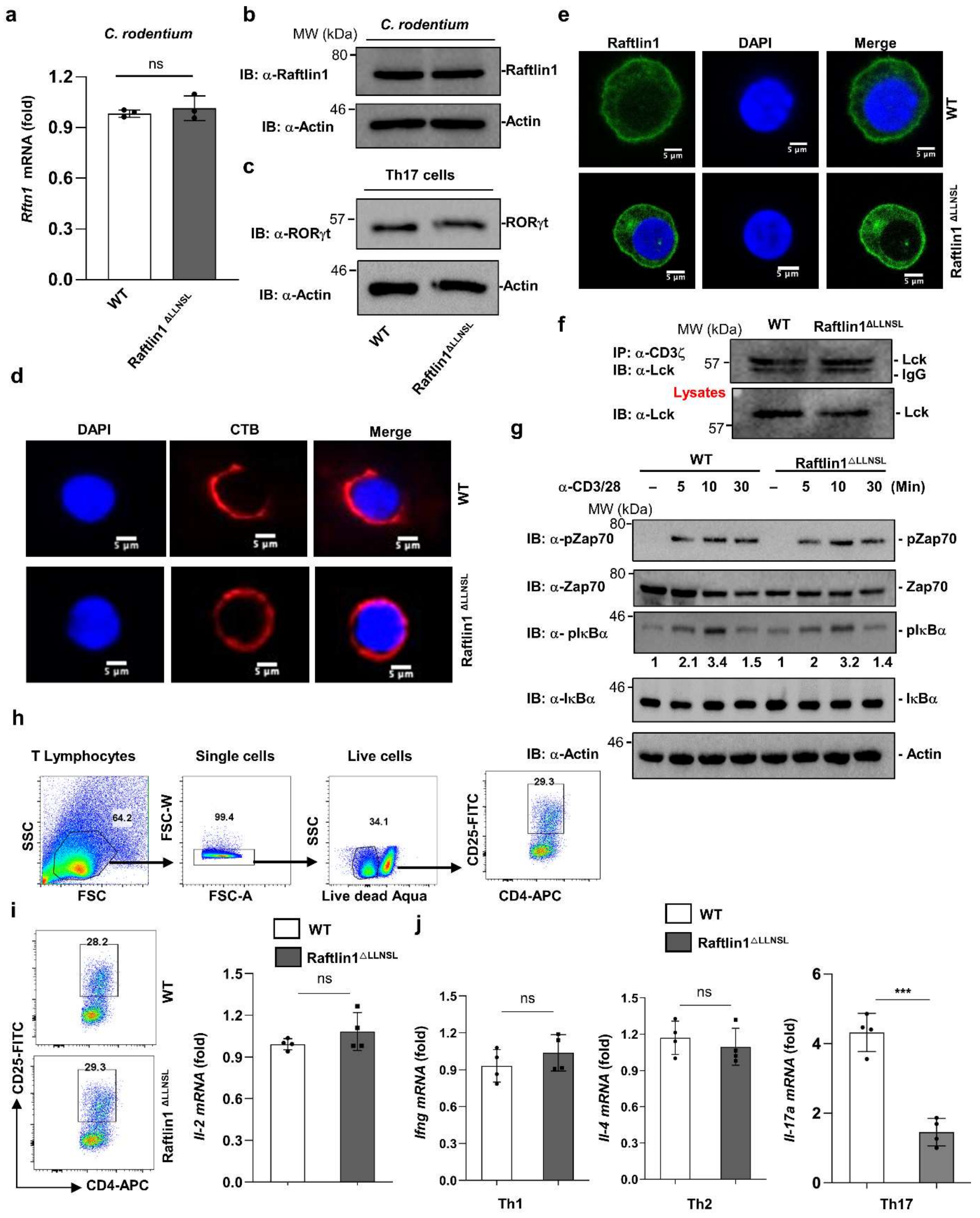

Supplementary Fig. 5: Lipid raft composition and TCR signaling in Raftlin1$^{\Delta LLNSL}$ T cells.

a-b, Expression of Raftlin1 in *C. rodentium* infected WT and Raftlin1$^{\Delta LLNSL}$ mice by real-time PCR and western blotting, respectively. n= 3 mice per group, p=0.508. **c**, Expression of RORγt in WT and Raftlin1$^{\Delta LLNSL}$ Th17 cells. **d,** CD4^{+} T cells were purified from WT and Raftlin1$^{\Delta LLNSL}$

mice and stimulated with anti-CD3 and anti-CD28 antibodies for 48 hours. The membrane GM1 was stained with cholera toxin B (CTB) to check lipid raft formation by microscopy. The image is representative of three independent experiments. Scale bars, 5 μm. **e**, Confocal microscopy analysis of the localization of Raftlin1 in WT and Raftlin1$^{\Delta LLNSL}$ in CD4^{+} T cells. The image is representative of three independent experiments. Scale bars, 5 μm. **f,** Lysates of activated CD4^{+} T cells were isolated from WT and Raftlin1$^{\Delta LLNSL}$ mice, immunoprecipitated with anti-CD3ζ antibody, and immunoblotted with anti-Lck antibody. **g,** CD4^{+} T cells from WT and Raftlin1$^{\Delta LLNSL}$ mice were stimulated with anti-CD3/anti-CD28 antibody for the indicated time. The cell lysates were prepared and analyzed by immunoblot with the indicated antibodies. **h**, Gating strategy for flow cytometry of CD4^{+} T cells from WT and Raftlin1$^{\Delta LLNSL}$ mice stained with Live-Dead Aqua, anti-CD4, and anti-CD25 antibodies. **i**, Expression of CD25 and *Il-2* on activated CD4^{+} T cells from WT and Raftlin1$^{\Delta LLNSL}$ mice; n=4 mice per group, p=0.244. **j**, CD4^{+} T cells isolated from WT and Raftlin1$^{\Delta LLNSL}$ mice and differentiated under Th1, Th2 and Th17 polarizing conditions. The expression of *Ifng*, *Il-4,* and *Il-17a* was analyzed by real-time PCR; n=4 mice per group, p=0.327 (*Ifng*), p=0.494 (*Il-4*), p=0.0001 (*Il-17a*). **b, c, f, g** Immunoblots are from one experiment representative of three independent experiments with similar results. Statistical significance was determined by unpaired Student's t-test (two-tailed) in **a, i, j** with $p < 0.05$ considered statistically significant. ***$p < 0.001$, ns- not significant, error bars are mean±SD. Source data are provided as a Source data file.

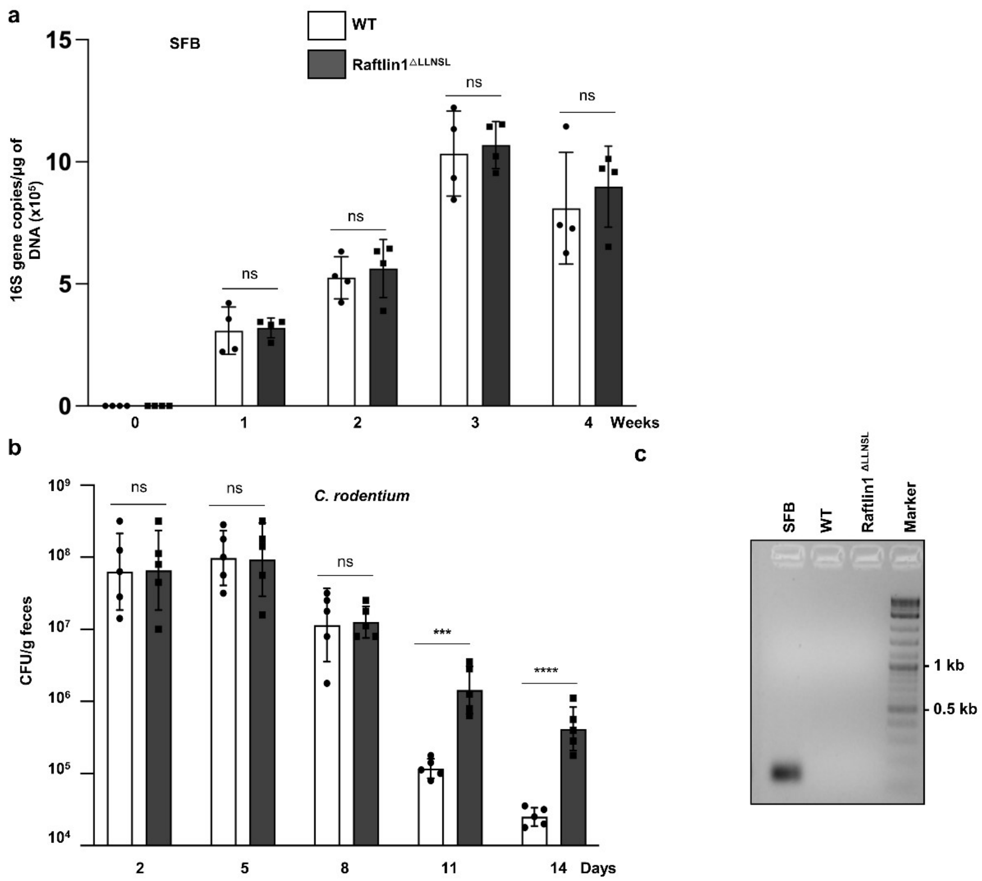

Supplementary Fig. 6: Analysis of SFB colonization and *C. rodentium* load in WT and Raftlin1$^{\Delta LLNSL}$ mice. a, Relative abundance of SFB in the feces of WT and Raftlin1$^{\Delta LLNSL}$ mice after gavage of feces from SFB monocolonized mice by quantifying SFB specific 16S using real-time PCR; n=4 biologically independent mice; p=0.83 (week 1), p=0.62 (week 2), p=0.74 (week 3), p=0.55 (week 4) **b**, Quantification of *C. rodentium* loads (CFU/g) in the stool of WT and Raftlin1$^{\Delta LLNSL}$ mice after the indicated time of the post-infection; n=5 mice per group; p=0.95 (day 2), p=0.94 (day 5), p=0.87 (day 8), p=0.0001 (day 11)**,** p=0.00003 (day 14)**. c,** the absence of SFB in our mice colony was confirmed by PCR using SFB-specific 16s primers. Data are from one experiment representative of three independent experiments with similar results. **a, b** Statistical significance was determined by unpaired Student's t-test (two groups) with $p < 0.05$ considered statistically significant. ***$p < 0.001$, ****$p < 0.0001$, ns- not significant, error bars are mean±SD. Source data are provided as a Source data file.

a

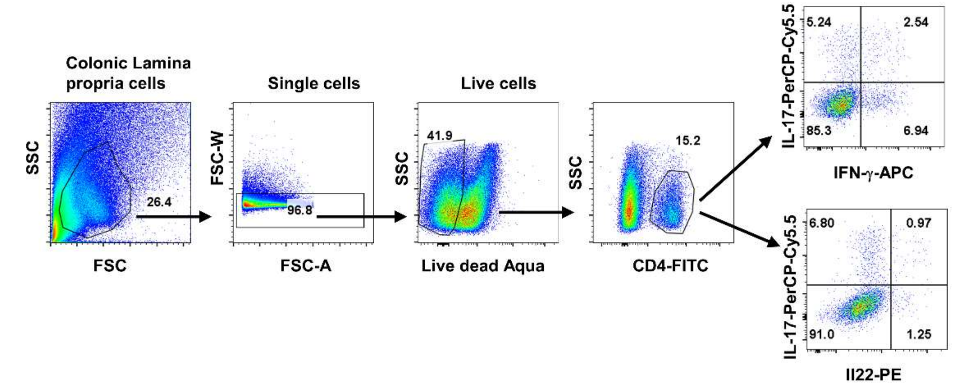

b

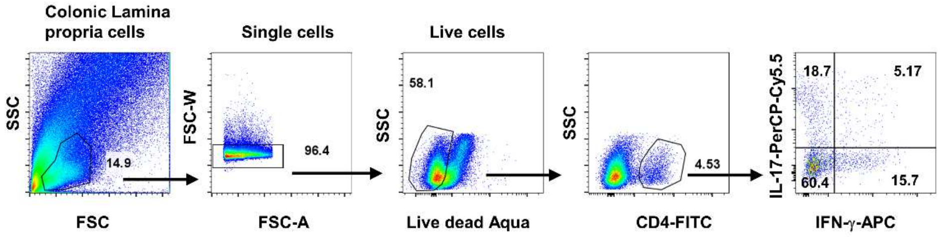

Supplementary Fig. 7: Flow cytometry gating strategy for the analysis of IL-17, IL-22, and IFN-γ in CD4$^+$ T cells of colonic lamina propria cells (Fig. 3e, f, 4g). a, Gating strategy of colonic lamina propria cells from WT and Raftlin1$^{\Delta LLNSL}$ mice stained with Live-Dead Aqua, anti-CD4, anti-IL-17, anti-IL22, and anti-IFN-γ antibodies, and analyzed by flow cytometry. **b**, Gating strategy of colonic lamina propria cells from *Rag1*$^{-/-}$ mice adoptively transferred CD4$^+$CD25$^-$CD45RB^{hi} cells from WT and Raftlin1$^{\Delta LLNSL}$ mice stained with Live-Dead Aqua, anti-CD4, anti-IL-17 and anti-IFN-γ antibodies and analyzed by flow cytometry. Data are from one experiment representative of three independent experiments with similar results.

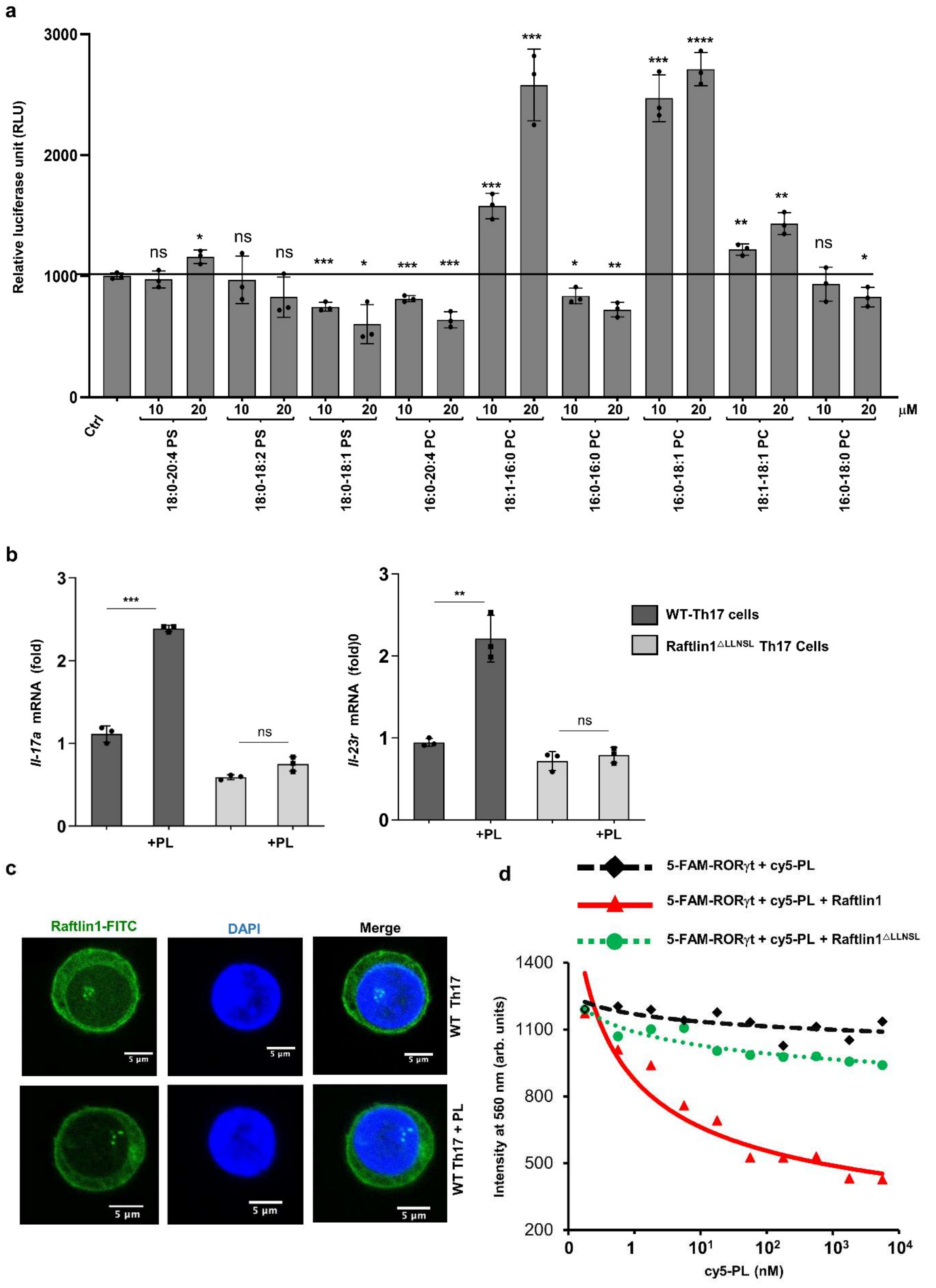

Supplementary Fig. 8: IL-17 promoter-luciferase reporter activity induced by lipids in Th17 cells. a, Jurkat T cells were cultured in lipid-depleted conditional media and transfected

with various combinations (below plot) of IL-17-promoter-driven luciferase plasmid (pGL4-mIL17p), the plasmid encoding Flag-RORγt along with Raftlin1 and cultured in the presence of various lipids (10μM or 20μM) for 16 h. The relative luciferase activity was measured to quantify the RORγt transcriptional activity. Results are presented in relative luciferase units (RLU). n= 3 biological replicates; (18:0-20:4 PS, 10μM p=0.57, 20μM p= 0.01), (18:0-18:2 PS, 10μM p=0.80, 20μM p= 0.15), (18:0-18:1 PS, 10μM p=0.0007, 20μM p= 0.014), (16:0-20:4 PS, 10μM p=0.0009, 20μM p= 0. 0009), (18:1-16:0 PS, 10μM p=0.0008, 20μM p= 0. 0008), (16:0-18:1 PS, 10μM p=0.0002, 20μM p<0. 0001), (18:1-18:1 PS, 10μM p=0.002, 20μM p= 0. 0014), (16:0-18:0 PS, 10μM p=0.46, 20μM p= 0. 02). **b**, Naive $CD4^+$ T cells were isolated from WT or Raftlin1$^{\Delta LLNSL}$ mice and differentiated under Th17 polarizing conditions followed by the addition of phospholipids (PL) 18:1-16:0-PC, 16:0-18:1-PC, and 18:1-18:1-PC at 48 h in lipid-depleted fresh media. The expression of *Il-17a* and *Il-23r* was measured by real-time PCR at 96 h. n= 3 biological replicates; p=0.00003 (*Il-17a*), p=0.0016 (*Il-23r*). **c,** Localization of Raftlin1 in WT Th17 cells cultured in the presence or absence of phospholipid by confocal microscopy. The image is representative of three independent experiments. Scale bars, 5 µm. **d**, Fluorescence emission intensity of 5-FAM labeled RORγt (5-FAM-RORγt) at 520 nm (Excitation = 470 nm) at increasing concentration of Cy5 labeled phospholipids (Cy5-PL) in the presence of GST-Raftlin1 (red triangles), GST-Raftlin1$^{\Delta LLNSL}$ (green dots), and control GST only (black dots). The concentrations of Cy5-PL (0-10^4 nM), 5-FAM-RORγt (5 nM), GST-Raftlin1 (10 nM), and GST-Raftlin1$^{\Delta LLNSL}$ (10 nM) were used. Data are from one experiment representative of three independent experiments with similar results. **a, b** Statistical significance was determined by unpaired Student's t-test (two-tailed) with $p < 0.05$ considered statistically significant. *p < 0.05, **p < 0.01, ***p < 0.001, ****p < 0.0001, ns- not significant, error bars are mean±SD. Source data are provided as a Source data file.

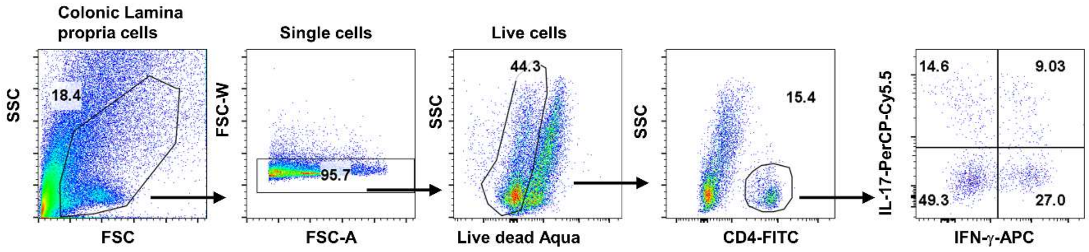

Supplementary Fig. 9: Flow cytometry gating strategy for the analysis of IL-17 and IFN-γ in CD4$^+$ T cells of colonic lamina propria cells (Fig. 5h). Gating strategy of colonic lamina propria cells from *Rag1$^{-/-}$* mice adoptively transferred phospholipid treated Th17 cells from WT and Raftlin1$^{\Delta LLNSL}$ mice stained with Live-Dead Aqua, anti-CD4, anti-IL-17, and anti-IFN-γ antibodies, and analyzed by flow cytometry. Data are from one experiment representative of three independent experiments with similar results.

Supplementary Table 1: List of lipids detected in Raftlin1 and RORγt immunoprecipitated samples from *C. rodentium* infected WT mice:

S. No	Lipids	IP: α-Raftlin1 (pmol/mg)	IP: α-RORγt (pmol/mg)	IP: IgG
1	1-stearoyl-2-arachidonoyl-sn-glycero-3-phospho-L-serine (**16:0-20:4-PS**)	56.41	50.93	0
2	1-stearoyl-2-linoleoyl-sn-glycero-3-phospho-L-serine (**18:0-18:2-PS**)	32.78	16.42	0
3	1-stearoyl-2-oleoyl-sn-glycero-3-phospho-L-serine (**18:0-18:1-PS**)	21.68	4.25	0
4	1-palmitoyl-2-arachidonoyl-sn-glycero-3-phosphocholine (**16:0-20:4-PC**)	40.39	21.14	0
5	1-oleoyl-2-palmitoyl-sn-glycero-3-phosphocholine (**18:1-16:0-PC**)	27.06	8.69	0
6	1,2-di-palmitoyl-sn-glycero-3-phosphocholine (**16:0-16:0-PC**)	21.85	5.21	0
7	1-Palmitoyl-2-oleoyl-sn-glycero-3-phosphocholine (**16:0-18:1-PC**)	20.22	18.54	0
8	1,2-dioleoyl-sn-glycero-3-phosphatidylcholine (**18:1-18:1-PC**)	63.19	11.51	0
9	1-palmitoyl-2-stearoyl-sn-glycero-3-phosphocholine (**16:0-18:0-PC**)	20.22	18.54	0

Supplementary Table 2: List of predicted Off-target genes

S.No	Genes	CFD Score
1	*Ccl9*	0.63
2	*Septin5*	0.13
3	*Cd9*	0.09
4	*Arsi*	0.09
5	*Tcaf3*	0.09
6	*Prx*	0.08
7	*Bmp10*	0.06
8	*Nsd1*	0.04
9	*Cdc5l*	0.03
10	*Prnp/Prn*	0.01
11	*Pdgfb*	0.00
12	*Myo16*	0.00
13	*Tns3*	0.00
14	*Gdnf*	0.00
15	*Kctd17*	0.00

Supplementary Table 3: PCR primers sequence of the predicted off-target genes

S.no	Genes	Primers
1	*Ccl9*	Fw:5'-CGTTTGATCCAGATCTTGAGGC-3'<br>Rv:5'-GGCTCAGCAGTTAAGAGCACTG-3'
2	*Septin5*	FW:5'-CCTCTTCTGCCTCGGGTTTT-3'<br>Rv:5'-CGGGTGAAGAGCAAGGAGAG-3'
3	*Cd9*	Fw:5'-AGCAATCTGTCACCTGGTCG-3'<br>Rv:5'-CAGTCTGTGTAGCCCTTGGG-3'
4	*Arsi*	Fw:5'-TGATCACCAGCACACCAGTC-3'<br>Rv:5'-GGCCACCGACCATTGTTAGA-3'
5	*Tcaf3*	FW: 5'-TGGCAGAAGTTGTGGAAGAGG-3'<br>Rv:5'-CTGCACAGACGCTATAGGGG-3'
6	*Prx*	Fw:5'-CACTCGACCTCTCTGGCAAG-3'<br>Rv:5'-GGGCTGACTCCAAGATCTTGT-3'
7	*Bmp10*	Fw:5'-ACCTCGGAGTAGCACCCTAG-3'<br>Rv:5'-GGGAGGGGAGAGTCTTCAGT-3'
8	*Nsd1*	Fw:5'-GCAGCAGCCATGTTTGTCAA-3'<br>Rv:5'-CGGGAAAAGTTCTCCAGGCT-3'
9	*Cdc5l*	Fw:5'-CAGATGGTTGGCTCAGTGGT-3'<br>Rv:5'-ATGGTGTCATCCCTCTGCAC-3'

Uncropped western blots and PCR agarose gels of Supplementary figures

Supplementary Fig. 1b

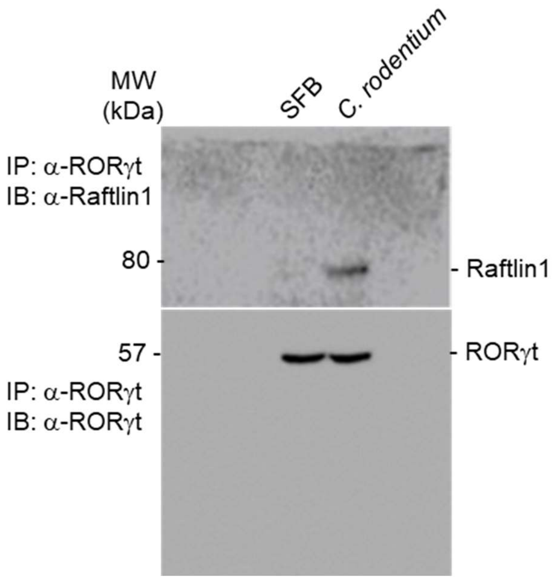

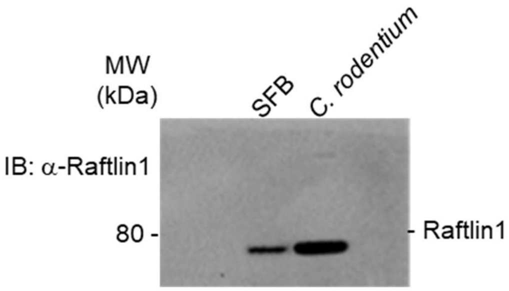

Supplementary Fig. 3a

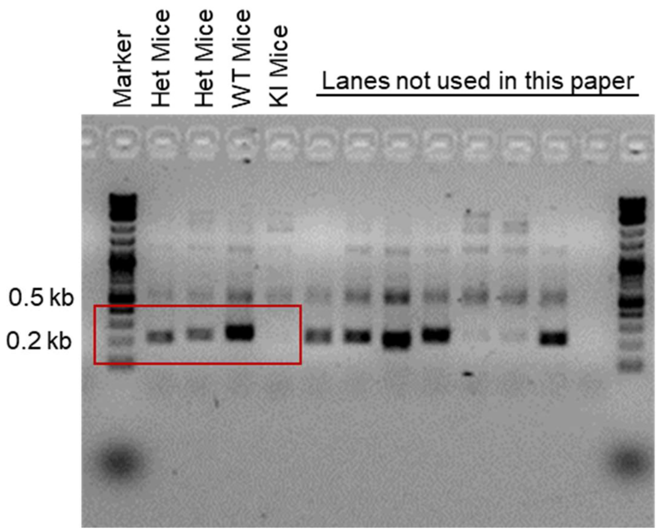

Supplementary Fig. 5b

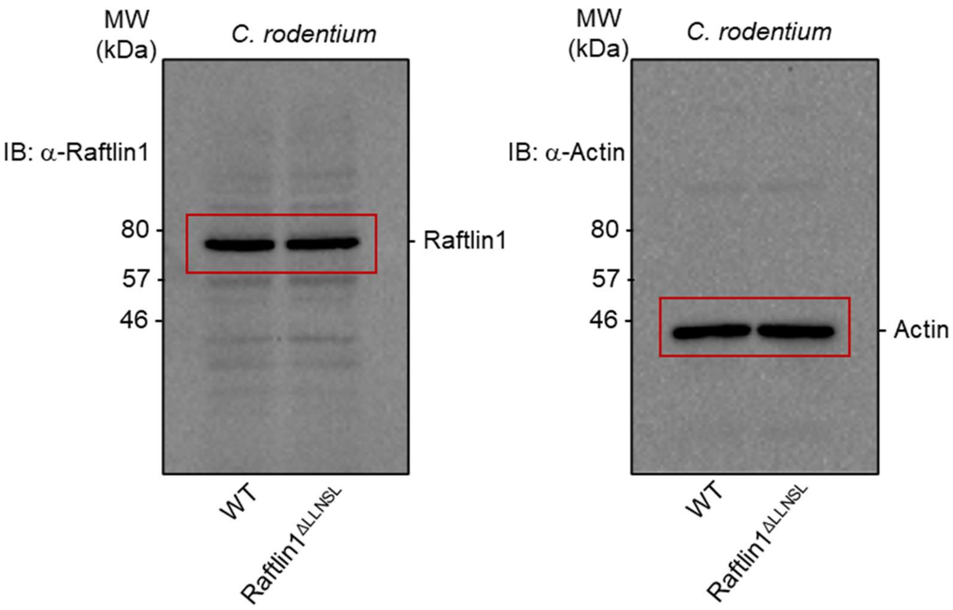

Supplementary Fig. 5c

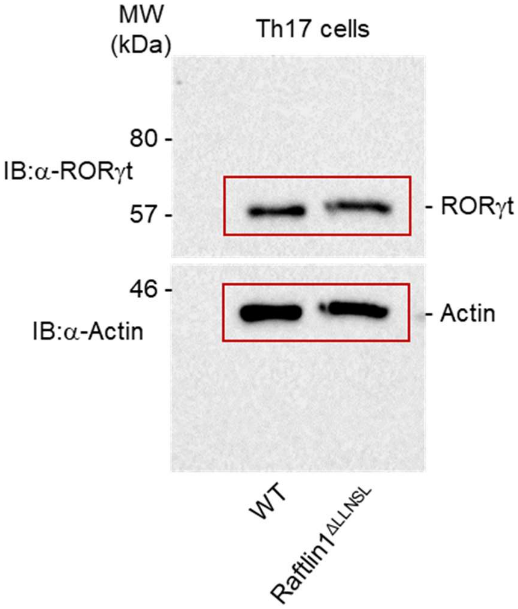

Supplementary Fig. 5f

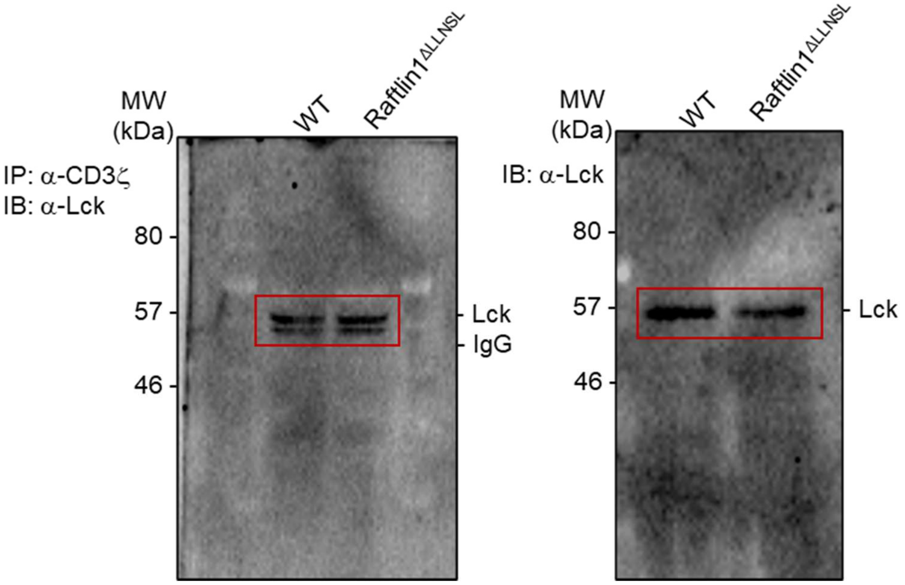

Supplementary Fig. 5g

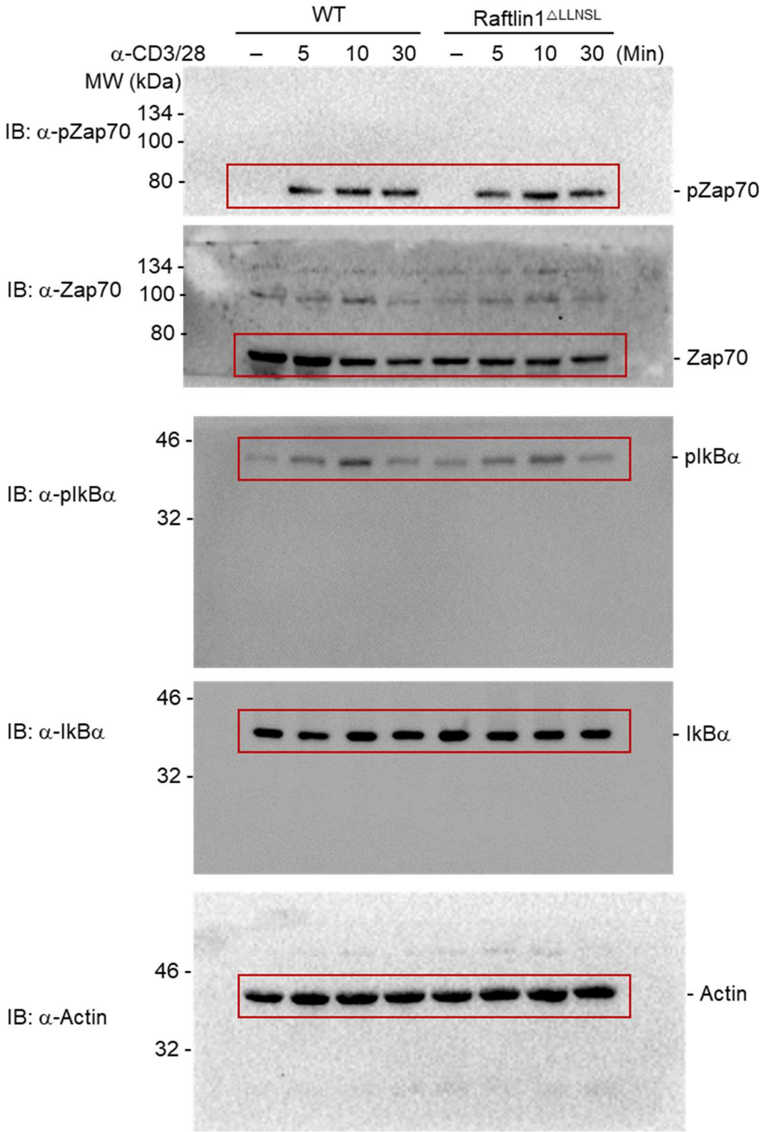

Supplement: Supplementary file 1 — Supplementary Information [file 41467_2023_40622_MOESM1_ESM.pdf]
