# Supplementary material for: RORγt-Raftlin1 complex regulates the pathogenicity of Th17 cells and colonic inflammation
Source: Nat Commun. 2023 Aug 17;14:4972. doi: 10.1038/s41467-023-40622-1 (PMC10435467; doi:10.1038/s41467-023-40622-1)
Supplement: Supplementary file 2 — Reporting Summary [file 41467_2023_40622_MOESM2_ESM.pdf]

nature portfolio

Corresponding author(s): K Venuprasad

Last updated by author(s): Jul 21, 2023

Reporting Summary

Nature Portfolio wishes to improve the reproducibility of the work that we publish. This form provides structure for consistency and transparency in reporting. For further information on Nature Portfolio policies, see our Editorial Policies and the Editorial Policy Checklist.

Statistics

For all statistical analyses, confirm that the following items are present in the figure legend, table legend, main text, or Methods section.

n/a	Confirmed	
☐	☒	The exact sample size (*n*) for each experimental group/condition, given as a discrete number and unit of measurement
☐	☒	A statement on whether measurements were taken from distinct samples or whether the same sample was measured repeatedly
☐	☒	The statistical test(s) used AND whether they are one- or two-sided<br>*Only common tests should be described solely by name; describe more complex techniques in the Methods section.*
☒	☐	A description of all covariates tested
☐	☒	A description of any assumptions or corrections, such as tests of normality and adjustment for multiple comparisons
☐	☒	A full description of the statistical parameters including central tendency (e.g. means) or other basic estimates (e.g. regression coefficient) AND variation (e.g. standard deviation) or associated estimates of uncertainty (e.g. confidence intervals)
☐	☒	For null hypothesis testing, the test statistic (e.g. *F*, *t*, *r*) with confidence intervals, effect sizes, degrees of freedom and *P* value noted<br>*Give P values as exact values whenever suitable.*
☒	☐	For Bayesian analysis, information on the choice of priors and Markov chain Monte Carlo settings
☒	☐	For hierarchical and complex designs, identification of the appropriate level for tests and full reporting of outcomes
☐	☒	Estimates of effect sizes (e.g. Cohen's *d*, Pearson's *r*), indicating how they were calculated

Our web collection on statistics for biologists contains articles on many of the points above.

Software and code

Policy information about availability of computer code

Data collection	Bio-Rad Image Lab 6.0.1, Eppendorf realplex 2.2, Nanodrop 2000c 1.4.2
Data analysis	FlowJo v10, AlphaFold2, HADDOCK2.4, GraphPad Prism 9, X!Tandem/TPP software suite, Microsoft Excel spreadsheet (Microsoft 365), ChemDraw Ultra 12.0, BioRender, ImageJ v1.53g

For manuscripts utilizing custom algorithms or software that are central to the research but not yet described in published literature, software must be made available to editors and reviewers. We strongly encourage code deposition in a community repository (e.g. GitHub). See the Nature Portfolio guidelines for submitting code & software for further information.

Data

Policy information about availability of data

All manuscripts must include a data availability statement. This statement should provide the following information, where applicable:

- Accession codes, unique identifiers, or web links for publicly available datasets
- A description of any restrictions on data availability
- For clinical datasets or third party data, please ensure that the statement adheres to our policy

The authors declare that the data supporting the findings of this study are available with the paper and its supplementary information files. All the data presented in graphs within the figures generated in this study are provided in the Source data excel files. The uncropped western blot images and PCR agarose gels are also provided in the Source data file. The uncropped western blot images and PCR agarose gels of supplementary data are also provided in the Supplementary

information. All data are available from the authors upon request.

Research involving human participants, their data, or biological material

Policy information about studies with human participants or human data. See also policy information about sex, gender (identity/presentation), and sexual orientation and race, ethnicity and racism.

Reporting on sex and gender	Sex and gender were not considered in the study design. However, sex information along with other variables (like disease location, family history of inflammatory bowel disease, etc.) was collected at the time of informed consent. Sex and gender were not considered relevant to the study as the focus of the study was to understand the molecular mechanism of colonic inflammation.
Reporting on race, ethnicity, or other socially relevant groupings	Healthy Control: Race- White (6), Asian (1), Black or African American (3); Ethnicity- Hispanic (2), Non-Hispanic/Latino (8)<br>Ulcerative colitis: Race - White (10), Black or African American (1); Ethnicity - Hispanic (3), Non-Hispanic/Latino (8)
Population characteristics	Healthy control: 10 cases; 2 Male (20 %), Age (55-59 years), Median age 56; 8 Female (80%), Age (45-66 years), Median age 56.5.<br>Active UC: 11 cases; 8 Male (72.7 %), Age (21-67 years), Median age 36; 3 Female (27.2 %), Age (19-53 years), Median age 40.25.
Recruitment	The study participants were recruited from the UT Southwestern Medical Center. Written informed consents were obtained from all patients that were recruited, without discrimination of any kind including gender, age, race, and nationality. Healthy controls were recruited which underwent colonoscopy for colorectal cancer screening/surveillance and showed no symptoms of inflammation. Active ulcerative colitis patients were recruited which had actively inflamed mucosa of the sigmoid colon based on endoscopic appearances.
Ethics oversight	The study protocol was approved by the Institutional Review Board of UT Southwestern Medical Center, Dallas, TX- 75390 (STU 112010-130).

Note that full information on the approval of the study protocol must also be provided in the manuscript.

Field-specific reporting

Please select the one below that is the best fit for your research. If you are not sure, read the appropriate sections before making your selection.

☒ Life sciences ☐ Behavioural & social sciences ☐ Ecological, evolutionary & environmental sciences

For a reference copy of the document with all sections, see nature.com/documents/nr-reporting-summary-flat.pdf

Life sciences study design

All studies must disclose on these points even when the disclosure is negative.

Sample size	Sample size was determined based on the common standard in the field. n>3 mice/group were used and all the animal experiments were repeated thrice. The number of independent samples used in the experiment are reported in the figure legends.
Data exclusions	We did not exclude any samples. Age and sex matched were used in all experiments. Reported in figure legends.
Replication	All experiments were repeated thrice or more. Reported in figure legends. All experimental results were reliably reproducible.
Randomization	Littermates were used as controls, wherever possible. We randomly chose mice from the same or different littermates for each experimental groups, independently of their gender and randomly chose the littermate control mice with same sex and age. Appropriate controls are used in each experiment.
Blinding	We did not blind to group allocation during experiments and data analysis. However, we carried out all the experiments and analyses without any prior biases and in an objective, rigorous scientific way. Conclusions were made based on statistical significance of the data.

Reporting for specific materials, systems and methods

We require information from authors about some types of materials, experimental systems and methods used in many studies. Here, indicate whether each material, system or method listed is relevant to your study. If you are not sure if a list item applies to your research, read the appropriate section before selecting a response.

Materials & experimental systems

n/a	Involved in the study
	[x] Antibodies
	[x] Eukaryotic cell lines
[x]	Palaeontology and archaeology
	[x] Animals and other organisms
[x]	Clinical data
[x]	Dual use research of concern
[x]	Plants

Methods

n/a	Involved in the study
[x]	ChIP-seq
	[x] Flow cytometry
[x]	MRI-based neuroimaging

Antibodies

Antibodies used	Anti-c-Myc (9E10, Santa Cruz biotechnology, sc-40, 1:1000), Anti-Flag (M2, Sigma Aldrich, F1804, 1:5000), Anti-RORγt (AFKJS-9, eBioscience, 14698882, 1:100), Anti-RORγt (O28-835, BD Bioscience, 562197, 1:500), Anti-GST (B14, Santa Cruz biotechnology, sc-138, 1:1000), Anti-β-actin (C4, Santa Cruz biotechnology, sc-47778, 1:1000), Anti-Phospho-Zap-70 (Tyr319)/Syk (Tyr352) (CST, 2701, 1:1000), Anti-Zap-70 (99F2, CST, 2705, 1:1000), Anti-Lck (3A5, Santa Cruz biotechnology, sc-433, 1:500), Anti-IκBα (CST, 9242, 1:2000), Anti-Raftlin (E11, Santa Cruz biotechnology, sc-514457, 1:1000), Anti-Raftlin-Alexa Fluor® 48 (E11, Santa Cruz biotechnology, sc-514457 AF488, 1:100), Anti-CD4-FITC (GK1.5, Biolegend, 100406, 1:200), Anti-CD4-PE (GK1.5, BD Bioscience, 553730, 1:200), Anti-CD4-APC (GK1.5, eBioscience, 17004181, 1:200), Anti-CD8-FITC (53-6.7, Biolegend, 100706, 1:200), Anti-CD25-FITC (PC61, Biolegend, 102006, 1:200), Anti-CD45RB-PE (16A, BD Bioscience, 553101, 1:200), Anti-Foxp3-PE (FJK-16s, eBioscience, 12577380, 1:50), Anti-CD44-FITC (IM7, BD Bioscience, 553133, 1:100), Anti-CD62L-APC-efluorTM 780 (MEL-14, eBioscience, 47062182, 1:200), Anti-IL17-PerCP-Cy5.5 (TC11, Biolegend, 506920, 1:20), Anti- IL-22-PE (Poly5164, Biolegend, 516404, 1:20), Anti-IFN-γ-APC (XMG1.2, BD Bioscience, 554413, 1:50), Anti-mouse CD3 (17A2, Biolegend, 100223), Anti-mouse CD28 (37.51, BioXCell, BE0015), Anti-mouse IFN-γ (R4-6A2, BioXCell, BE0054), anti-IL4 (11B11, Biolegend, 504102), Clean-Blot™ IP detection reagent (HRP) (Themo Fisher Scientific, 21230, 1:200), Anti-mouse IgG for IP (HRP) (abcam, ab131368, 1:5000)
Validation	All antibodies used in the study were obtained from commercial source and validated by the manufacturers (see manufacturer's website). Additionally, most antibodies (Anti-c-Myc, Anti-Flag, Anti-RORγt, Anti-GST, Anti-β-actin, Anti-CD4-FITC, Anti-CD4-PE, Anti-CD4-APC, Anti-CD8-FITC, Anti-CD25-FITC, Anti-CD45RB-PE, Anti-Foxp3-PE, Anti-IL17-PerCP-Cy5.5, Anti-IFN-γ-APC) were validated as demonstrated in our previous publication (Kathania et al., Nat immunol. 2016, Singh et al., Nat comm, 2018, Kathania et al., J immunol. 2015, Theivanthiran at al., Sci signal. 2015)

Eukaryotic cell lines

Policy information about cell lines and Sex and Gender in Research

Cell line source(s)	293T (ATCC, CRL-3216™), Jurkat, Clone E6-1 (ATCC, TIB-152™)
Authentication	All the cell lines used in the study were obtained from commercial source and authenticated by the manufacturers (see manufacturer's website).
Mycoplasma contamination	Tested negative for mycoplasma contamination.
Commonly misidentified lines (See ICLAC register)	None of the cell lines used in this study are listed in the ICLAC database.

Animals and other research organisms

Policy information about studies involving animals; ARRIVE guidelines recommended for reporting animal research, and Sex and Gender in Research

Laboratory animals	C57BL/6, Rag1-/-, Raftlin1-LLNSL of 8-10 weeks of age (male and female both) were used in this study. Genotypes were verified by PCR analysis. Mice were housed in a pathogen free facility at the UT Southwestern Medical Center, according to the Institutional Animal Care and Use Committee (IACUC). Standard conditions of dark/light cycle, ambient temperature and humidity were used as provided by IACUC.
Wild animals	No wild animals were used in this study.
Reporting on sex	Both male and female mice were used in this study.
Field-collected samples	No field-collected samples were used in this study.
Ethics oversight	All animal procedures were performed in accordance with the approved protocols by the Institutional Animal Care and Use Committee (IACUC) of UT Southwestern Medical Center, Dallas, TX 75390. IACUC No.: 2019-102734, 2019-102735.

Note that full information on the approval of the study protocol must also be provided in the manuscript.

Flow Cytometry

Plots

Confirm that:

- [x] The axis labels state the marker and fluorochrome used (e.g. CD4-FITC).
- [x] The axis scales are clearly visible. Include numbers along axes only for bottom left plot of group (a 'group' is an analysis of identical markers).
- [x] All plots are contour plots with outliers or pseudocolor plots.
- [x] A numerical value for number of cells or percentage (with statistics) is provided.

Methodology

Sample preparation	Single-cell suspensions were prepared from spleen, mLN, thymus, payers patch (PP) and colon as described in methods, and stained with viability dye (live/dead aqua). The cells were further stained with antibodies against CD4, CD8, CD25, CD44 and CD62L, fixed and permeabilized in BD Fix/Perm buffer, and stained intracellularly with anti-IL-17, anti -IL-22 anti -IFN-g. For CD4+CD25–CD45RBhi T cell sorting, single-cell suspensions from the spleen were enriched for CD4+ T cells with Mouse CD4+ T Cell Isolation kit according to the manufacturer's instructions. Cells were stained with antibodies against CD4, CD25, and CD45RB and sorted as live CD4+CD25–CD45RBhi cells for adoptive transfer experiments.
Instrument	Samples were run on a BD FACS CantoTM.
Software	Analysis of the samples was performed with FlowJo v10 software.
Cell population abundance	Single-cell suspensions were prepared from spleen, mLN, thymus, payers patch (PP) and colon of mice and stained with respective antibodies, and analyzed by flow cytometry. The abundance of cell population are collected by FlowJo software. In gating strategy presented in the manuscript, the cell populations involved in the calculation of cell abundance are labeled.
Gating strategy	1. SSC vs. FSC gating to exclude debris. 2. FSC-H vs. FSC-A gating to exclude doublets. 3. 7-AAD/Live-dead aqua vs. FSC-A gating to exclude dead cells. 4. CD4, CD8, CD25, FoxP3, CD45RB, IL-17, IL-22, IFNg to quantify positive cells against respective antibodies. Boundaries for Gate 4 were based on Isotype control. The gating strategy of different cell populations of main and supplementary figures are reported in the supplementary Information.

- [x] Tick this box to confirm that a figure exemplifying the gating strategy is provided in the Supplementary Information.
